# Bioturbation as a key driver behind the dominance of Bacteria over Archaea in near-surface sediment

**DOI:** 10.1038/s41598-017-02295-x

**Published:** 2017-05-25

**Authors:** Xihan Chen, Thorbjørn Joest Andersen, Yuki Morono, Fumio Inagaki, Bo Barker Jørgensen, Mark Alexander Lever

**Affiliations:** 1 Center for Geomicrobiology, Department of Bioscience, Aarhus University, 8000 Aarhus, Denmark; 2 Department of Geosciences and Natural Resource Management, University of Copenhagen, 1350 Copenhagen, Denmark; 3 Kochi Institute for Core Sample Research, Japan Agency for Marine-Earth Science and Technology (JAMSTEC), Nankoku, Kochi 783-8502 Japan; 4 Institute of Biogeochemistry and Pollutant Dynamics, Department of Environmental Systems Science, ETH Zürich, 8092 Zürich, Switzerland

## Abstract

The factors controlling the relative abundances of Archaea and Bacteria in marine sediments are poorly understood. We determined depth distributions of archaeal and bacterial 16S rRNA genes by quantitative PCR at eight stations in Aarhus Bay, Denmark. Bacterial outnumber archaeal genes 10–60-fold in uppermost sediments that are irrigated and mixed by macrofauna. This bioturbation is indicated by visual observations of sediment color and faunal tracks, by porewater profiles of dissolved inorganic carbon and sulfate, and by distributions of unsupported ^210^Pb and ^137^Cs. Below the depth of bioturbation, the relative abundances of archaeal genes increase, accounting for one third of 16S rRNA genes in the sulfate zone, and half of 16S rRNA genes in the sulfate-methane transition zone and methane zone. Phylogenetic analyses reveal a strong shift in bacterial and archaeal community structure from bioturbated sediments to underlying layers. Stable isotopic analyses on organic matter and porewater geochemical gradients suggest that macrofauna mediate bacterial dominance and affect microbial community structure in bioturbated sediment by introducing fresh organic matter and high-energy electron acceptors from overlying seawater. Below the zone of bioturbation, organic matter content and the presence of sulfate exert key influences on bacterial and archaeal abundances and overall microbial community structure.

## Introduction

Archaeal and bacterial populations in marine sediments account for a significant fraction of global living biomass^1, 2^. Members of both domains drive globally important biogeochemical processes, e.g. Archaea perform methanogenesis and Bacteria perform sulfate reduction. In recent years, novel insights into the metabolism of uncultivated marine sediment Archaea and Bacteria have emerged from studies involving fluorescence-*in-situ*-hybridization (FISH), single-cell genomics, and metagenomics^3–5^. Yet, much remains to be learned^6^, and it is currently not even known which variables drive the relative abundances of Archaea and Bacteria in marine sediments.

Depth profiles of archaeal and bacterial populations frequently show a large scatter^7–9^, possibly due to vertical heterogeneity in sediment geochemistry and lithology^10^. Methods for quantification of microbes, e.g. FISH, quantitative PCR (qPCR), or lipid biomarker analyses, often show limited agreement between each other^7, 11–13^, suggesting inherent methodological biases. Limited agreement is also common when results from different FISH protocols, nucleic acid extraction methods, and lipid biomarker analytical methods are compared amongst each other, presumably due to variations in extraction efficiency^14–16^, cell labeling efficiency^7^, PCR primer biases^14, 17^, and instrument sensitivities^18, 19^. In addition, many standard DNA and lipid analyses rely on bulk extraction methods that include intra- and extracellular DNA and lipid pools. DNA outside living cells accounts for ≥10–90% of total DNA^14^, and extracellular lipids may have long half-lives, in particular those of archaeal origin^20^. Significant quantitative biases due to inclusion of extracellular DNA and lipids from outside of living cells are thus likely.

Despite uncertainties associated with each quantification method, almost all studies suggest that both archaeal and bacterial abundances decrease with depth in marine sediments^7–12, 14, 15^. Most studies, moreover, indicate that Bacteria dominate^8, 21–23^ or occur in equal proportions to Archaea in marine subsurface sediments^7, 9, 12^, and that Bacteria clearly dominate over Archaea in surface sediments^22, 24–26^. Reasons for the dominance of Bacteria in surface sediments are not known, but vertical changes in relative abundances might provide clues. Lipp *et al*.^15^ measured a dramatic shift in dominance from bacterial to archaeal intact polar lipids (IPLs) in the top 10 cm of sediments on the Peru Margin, and interpreted this shift to confirm the notion of Valentine^27^, who had proposed that Archaea are better adapted to longterm low-energy stress than Bacteria. Based on Catalyzed-Reporter deposition-FISH (CARD-FISH), Molari *et al*.^24^ documented a clear decrease in Bacteria-to-Archaea Ratios (BAR) in the top 10–15 cm of sediment in the southern Adriatic Sea, and attributed this shift to vertical changes in sediment water content and grain size.

Here we examine the relative abundances of archaeal and bacterial 16S rRNA genes (abbreviated as “16S genes” hereafter in the text) from the seafloor down to 11 meters below seafloor (mbsf) at eight stations (stations M1, M5, M21, M22A, M24, M26, M27A and M29A; Supplementary Fig. 1) in Aarhus Bay, Denmark. Intact surface sediments were recovered using 1-m Rumohr Lot cores, while deeper layers were sampled by 6–12 m long gravity cores. With the exception of a freshwater peat layer (M1) and a glacial sand layer (M21) at the bottom of cores from two stations, all sediments consisted of Holocene marine mud. We extracted DNA using a method that includes an initial wash step to separate soluble DNA (sDNA) that is dissolved in sediment porewater or adsorbed to sediment matrices from non-soluble (nsDNA) particulate pools that include DNA from living cells^14^. Archaeal and bacterial 16S gene abundances were then quantified in nsDNA extracts by qPCR using domain-specific primers. Variations in the depth of the sulfate-methane transition zone (SMTZ) and the presence of free methane gas between stations allowed us to investigate links between archaeal and bacterial abundance and the distribution of sulfate reducing and methanogenic activity and free methane gas.

Initial results showed that the biggest shifts in archaeal and bacterial 16S gene abundances occurred in the top 20 cm of sediment rather than across the SMTZs or due to presence of free methane gas. We hypothesized these shifts to be linked to macrofaunal activity, and thus compared trends in archaeal and bacterial 16S gene abundances in surface sediments to patterns in macrofaunal activity. We inferred the vertical extent of bioirrigation, i.e. the movement of overlying seawater into sediment by macrofauna during feeding and respiratory activities^28^, from porewater concentration profiles of sulfate, dissolved inorganic carbon (DIC), and δ^13^C-DIC. We calculated the vertical extent of sediment reworking, i.e. the vertical mixing of sediment due to macrofaunal deposit feeding and burrowing activities^28^, based on sedimentation rates and depth profiles of radionuclides (unsupported ^210^Pb (^210^Pb_unsupp_), ^137^Cs)^29, 30^. We, moreover, compared vertical profiles of 16S gene abundances with those of solid-phase total organic carbon (TOC) and total nitrogen (consisting to >95% of organic N), ratios of TOC:TN (called C:N ratios from now on), δ^13^C-TOC, and δ^15^N-TN. Hereby TOC and TN served as proxies for bulk organic matter (OM) content, whereas C:N, δ^13^C-TOC, and δ^15^N-TN were used as proxies for bulk OM composition.

Our results indicate that the activities of benthic macrofauna are a critical, yet widely overlooked determinant of archaeal and bacterial abundances in marine surface sediments. Mechanisms by which sediment reworking and bioirrigation may affect abundances of Archaea and Bacteria and overall microbial community structure are discussed, in addition to likely drivers behind microbial community structure in sediments below the bioturbation zone.

## Results

### Trends in archaeal and bacterial 16S gene abundances vs. sediment depth

**Figure 1:**
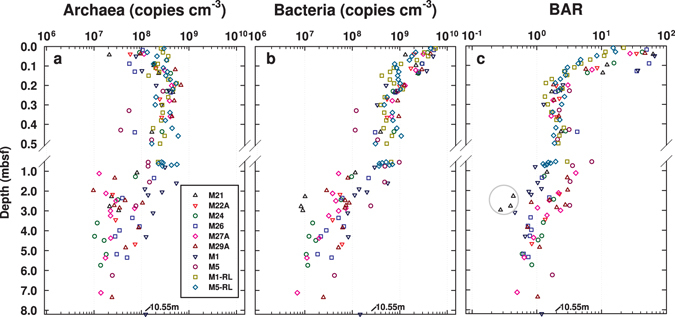
(**a**) Archaeal and (**b**) bacterial 16S rRNA gene copy numbers (copies cm^−3^ wet sediment) and (**c**) BARs across a transect of eight stations in Aarhus Bay. The gray circle in (**c**) marks sediment samples from an organic-poor glacial sand layer at the bottom of M21. The SMTZ was reached at all stations except M21 and M22A, and varied greatly in depth and width (M24: 3.36–4.31 mbsf; M26: 2.04–3.78 mbsf; M27A: 2.62–2.90 mbsf; M29A: 2.20–2.75 mbsf; M1: 1.40–1.60 mbsf; M5: 0.47–0.58 mbsf). Free methane gas was detected at five stations (M26: ≥7.04 mbsf, M27A: ≥4.70 mbsf; M29A: ≥4.45 mbsf; M1: ≥4.00 mbsf; M5: ≥1.00 mbsf). Gravity and Rumohr cores were taken from M21, M22A, M24, M26, M27A, M29A, and M5 in May 2010, and from M1 in October 2009. Additional, increased sample-resolution Rumohr cores from M1 (M1-RL) and M5 (M5-RL) were obtained in March 2012.

Depth distributions of archaeal and bacterial 16S gene abundances show consistent trends across all eight stations (Fig. 1). Bacterial greatly outnumber archaeal 16S genes in the top 10–20 cm. BARs are typically highest in the top 0–4 cm, where they range from 10:1 to 60:1, and then decrease due to a decrease in bacterial and concomitant increase in archaeal gene abundance down to ~20 cm. Below 20 cm, both archaeal and bacterial 16S gene abundances gradually decrease with depth. Bacterial decreases in 16S gene abundances are slightly steeper than those for Archaea, as is evident from mean BARs of ~2:1 at 20 cm depth which gradually decrease to ~1:1 at 3 mbsf. These overall trends are consistent throughout the Holocene mud layers across all stations, and suggest that the biggest changes in BARs occur within a small, uppermost part of the sulfate reduction zone, and are thus not driven by sulfate concentrations, or the presence of free methane gas (Supplementary Table 1). BARs are also similar between Holocene mud (0–7.4 mbsf) and an underlying peat layer (below 10.5 mbsf) at M1, but are interestingly lowest in a glacial sand layer at M21, where archaeal 16S genes conspicuously outnumber bacterial 16S genes by factors of 2 to 4.

When the 8 Aarhus Bay stations are examined individually, the above trends are even more clear, due to very low data scatter between adjacent depths from the same stations (Supplementary Fig. 2 and next section). Bacterial outnumber archaeal 16S genes by at least one order of magnitude at the sediment surface. Archaeal 16S gene abundances increase and bacterial 16S gene abundances decrease downward from the sediment surface to ~20 cmbsf. Below ~20 cmbsf, both archaeal and bacterial 16S gene abundances decrease, with bacterial typically exceeding archaeal 16S genes down to the SMTZ, and reaching similar values in bacterial and archaeal gene copy numbers below. The exception is again M21, where archaeal outnumber bacterial 16S genes in sulfate-rich sediment associated with organic-poor glacial sand. Moreover, small local increases in 16S gene abundances of Archaea and/or Bacteria are present in the SMTZ at five out of the six stations where the SMTZ was reached by coring.

### Fine-scale depth profiles in surface sediments

**Figure 2:**
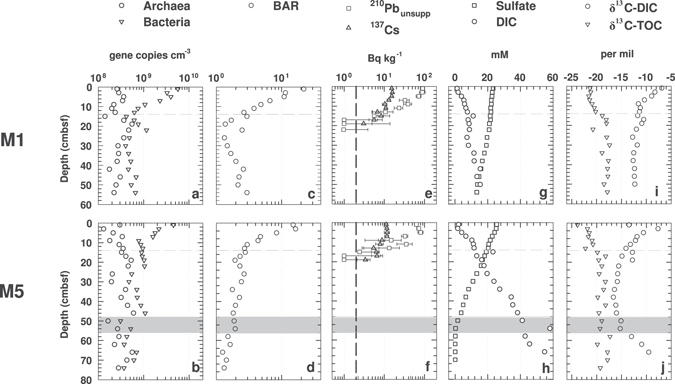
Fine-scale microbial abundance and geochemical profiles of surface sediments at M1 (M1-RL, top row of panels) and M5 (M5-RL, bottom row of panels). (**a,b**), abundances of archaeal and bacterial 16S genes; (**c,d**), BARs; (**e,f**) mean ^210^Pb_unsupp_ and ^137^Cs values ± standard deviation (SD) - the detection limit of ^210^Pb_unsupp_ and ^137^Cs is indicated by the dashed black line; (**g,h**), concentrations of sulfate and DIC; (**i,j**), δ^13^C-DIC and -TOC. The horizontal grey dashed lines indicate 0.14 mbsf, which is the minimum depth of macrofaunal sediment reworking based on ^210^Pb_unsupp_. The shaded grey area at M5 marks the interval of the SMTZ.

Given that sedimentation rates vary between stations in Aarhus Bay, e.g. M1 is in an area of Aarhus Bay with net erosion^31^, whereas stations M24, M26, M27A, M29A, and M5 are subjected to variable but significant rates of net sedimentation (0.86 to 1.11 mm yr^−1^ ^32^; note: no data are available for M21 or M22A), the high similarity in BAR depth profiles is unlikely to be driven by sedimentation alone or sedimentation-related rates of OM deposition. The rates and depth profiles of microbial respiration in surface sediments are also too different^33^, and seasonal changes in microbial respiration too high^34^ to explain the observed congruence in BAR depth profiles across sites and sampling dates. We hypothesize that the activity of macrofauna inhabiting the top layer of sediment at all Aarhus Bay sites has a ‘homogenizing’ effect that causes similar depth profiles in bacterial and archaeal abundances across sites. To assess this hypothesis, we compared the high depth-resolution qPCR data on Rumohr Lot cores from M1 and M5 (labeled M1-RL and M5-RL in Fig. 1) to a range of geochemical data that provide clues to the depths of macrofaunal reworking, OM input, and bioirrigation (Fig. 2, Supplementary Fig. 4).

Both Rumohr cores show the characteristic decrease in bacterial and concomitant increase in archaeal 16S genes in the top 20 cmbsf (Fig. 2a,b). Similarly, the BARs show a clear and consistent decrease, from ~20:1 down to ~1:1 at M1, and from ~15:1 down to ~2:1 at M5 (Fig. 2c,d). Closer inspection suggests that the stabilization of BARs occurs slightly above 20 cmbsf. At M1, BARs stabilize at ~1:1 at ~16 cmbsf, whereas BARs at M5 stabilize at ~2:1 at ~12 cmbsf.

**Figure 3:**
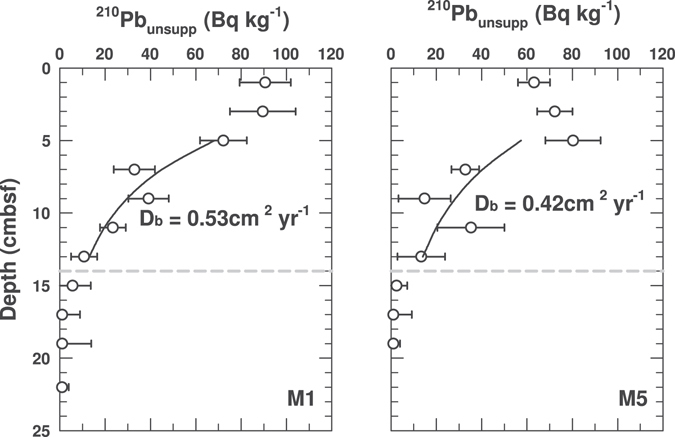
Modeled sediment mixing coefficients, D_b_, based on depth profiles of ^210^Pb_unsupp_ in the depth interval from 4–14 cmbsf at M1 and M5. Error bars indicate the standard deviation of ^210^Pb_unsupp_ within each depth sampled. The horizontal grey dashed lines indicate the minimum depth of macrofaunal sediment reworking (0.14 mbsf).

Highly similar radionuclide profiles indicate similar depth distributions of macrofaunal sediment reworking at M1 and M5. ^210^Pb_unsupp_ concentrations are uniformly high from the sediment surface to 6 cm depth (Figs 2e,f and 3), indicating high rates of vertical sediment mixing over time scales that are much shorter than the half-life of the tracer. Below 6 cmbsf, ^210^Pb_unsupp_ values drop considerably but remain above the detection limit down to 14 cmbsf, indicating continued albeit reduced vertical sediment mixing. Calculated particle mixing coefficients (D_b_) for the interval from 6 to 14 cm are 0.53 cm yr^−1^ at M1 and 0.42 cm yr^−1^ at M5 (Fig. 3). Below 14 cmbsf, ^210^Pb_unsupp_ is below detection, indicating low or no vertical sediment mixing. The ^210^Pb_unsupp_ values strongly co-vary with ^137^Cs values, which are lower than the ^210^Pb_unsupp_ at the sediment surface but higher at depth, remaining above detection to ~18 cmbsf (Fig. 2e,f), in accordance with the longer half-life (22.3 years for ^210^Pb and 30.2 years for ^137^Cs). Overall, the radionuclide data are consistent with visual observations of bioturbation made on Rumohr cores at M1 and M5, which indicate the presence of a heavily mixed and oxidized ‘surface mixed layer’ (SML; to 4 ± 1 cmbsf), and a’deep mixed layer’ (DML) that is dark grey but contains light brown streaks to ~10 cmbsf at M1, and to 12–20 cmbsf at M5; these streaks indicate occasional transport of oxidized surface sediment to deeper layers that are otherwise grey due to the production of iron(II) sulfides under reducing conditions (more info under ‘Core Descriptions (2012)’ in Supplementary Methods).

Porewater concentrations of DIC and sulfate at M1 and M5 indicate strong bioirrigation of surface sediment (Fig. 2g,h). DIC concentrations remain around seawater values (~2 mM) in the SML, consistent with high rates of porewater exchange between sediments and overlying seawater, and then increase, reaching 8.6 and 16 mM at 20 cm depth at M1 and M5, respectively. These increases in DIC concentrations indicate that, unlike in the SML, macrofaunal bioirrigation rates in the DML are insufficient to dilute out the DIC signal produced by microbial mineralization of OM. DIC concentration increases are steepest from 4 to 8 cmbsf at M1 and from 4 to 10 cmbsf at M5 and level off below 8 and 10 cmbsf, respectively, indicating a depth-related decrease in microbial CO_2_ production. The steeper increase in DIC concentrations with depth at M5 reflects the higher rates of microbial mineralization of OM at this station compared to M1.

Sulfate concentrations at M1 and M5 decrease with sediment depth, indicating microbial sulfate reduction. At M1, sulfate concentrations show a steady decrease from the seafloor downward, with a slight inflection in the sulfate profile at ~18 cmbsf. This inflection suggests input of sulfate-rich seawater by bioirrigation or significant production of sulfate by re-oxidation of reduced sulfur compounds with seawater-derived electron acceptors, e.g. O_2_ or nitrate, to this depth. This interpretation is supported by the concentration gradient of sulfate in the SML and DML. Despite sulfate reduction rates being the highest in the SML and DML^33, 34^, the sulfate concentration gradient is less steep than in the underlying sediment, where sulfate reduction rates are orders of magnitude lower^33^ and only a low sulfate flux is required to account for the entire depth-integrated reduction of sulfate^32^. At M5, sulfate concentrations are nearly constant in the SML, suggesting very high rates of bioirrigation that replace nearly all sulfate that is consumed by sulfate reduction. Below 4 cmbsf, sulfate concentrations at M5 decrease more steeply than at M1, consistent with higher microbial respiration rates at M5. The sulfate concentration profile below 4 cmbsf is near-linear, in spite of the highest measured sulfate reduction rates at M5 occurring in the top 10 cmbsf^33^; as at M1, the sulfate concentration profile in the SML and DML of M5 thus indicates a significant input of sulfate by bioirrigation. Reflecting the higher rates of OM remineralization at M5, sulfate is depleted at 56 cmbsf, while at M1 sulfate concentrations are still >10 mM at this depth. Methane concentrations increase slightly but remain in the micromolar range throughout the bioturbated surface interval and sulfate zones at both stations, and only show strong increases below the SMTZ (Supplementary Fig. 3a,b).

The δ^13^C-isotopic compositions of DIC and TOC show clear depth-related trends that are consistent with the radionuclide and porewater concentration data (Fig. 2i,j). The δ^13^C-DIC at both stations approaches seawater DIC (~0‰) at the sediment surface and decreases with depth, suggesting an increasing proportion of DIC produced by organic carbon diagenesis. The δ^13^C-DIC at M1 is approximately −6.9‰ in surface sediment, decreases steeply to −11.6‰ at 10 cmbsf, and then decreases only slightly to ~ −12‰ below 20 cmbsf. Apart from an abnormal δ^13^C-DIC of −13.5‰ in the top 2 cm, which can be considered an outlier, the δ^13^C-DIC depth profile at M5 shows high similarity to that at M1. Values decrease steeply from −7.7‰ to −14‰ between 2 to 14 cmbsf, and then continue to decrease more gradually, stabilizing around −16‰ at 20 cm and below. Consistent with the formation of ^12^C-enriched methane by CO_2_ reduction at M5, DIC progressively becomes ^13^C-enriched in the methane zone.

The δ^13^C-TOC shows a similar trend at both stations. The δ^13^C-TOC is lowest in surface sediments, ranging from −22 to −21‰ in the top 8 cm of M1 and from −23 to −22.5‰ in the top 4 cm of M5. Below, the δ^13^C-TOC increases, and then stabilizes around −18.5 to −17‰ below 16 cmbsf at M1 and below 20 cmbsf at M5. These changes in δ^13^C-TOC indicate a possible shift in the predominant OM source from surface sediment to the bottom of the bioturbation zone (also see next section). By comparison, the TOC contents in surface sediments do not show consistent trends across stations (Supplementary Fig. 3c,d). At M1, TOC decreases from ~3.8% in surface sediments to ~3% at 20 cmbsf, and fluctuates between 2.5 to 3.7% below. At M5, TOC increases from ~2.5% at the seafloor to ~4% at 20 cmbsf, and fluctuates between ~2.9 and 4.7% below.

To check for relationships between relative abundances of archaeal and bacterial 16S genes and sediment water content or grain size, we also measured sediment porosity (Supplementary Fig. 3e,f) and density (Supplementary Fig. 3g,h). While porosity decreased and density increased in the top 10 cm at M1, there was no significant change in porosity or density in surface sediments at M5. Based on the lack of a consistent relationship to archaeal and bacterial 16S gene abundances, we conclude that neither sediment water content nor grain size are drivers of archaeal and bacterial 16S gene abundances in Aarhus Bay.

### Comparison to Non-Bioturbated Station in Aarhus Harbor

Surface sediments at a non-bioturbated station in Aarhus Harbor (AHB) display a different trend in bacterial and archaeal abundances compared to the stations in Aarhus Bay (Supplementary Fig. 4). Here the abundances of archaeal and bacterial 16S genes co-vary in surface sediment, with both showing overall decreases with depth. The BAR profile fluctuates between 3:1 and 9:1 and decreases only slightly with depth. Compared to the stations in Aarhus Bay, BARs at AHB are consistently lower than at M21, M22A, M24, M26, M27A, M29A, and M1. Compared to M1-2012 and M5-2012, BARs at AHB are clearly lower in the top 6 cmbsf, but display similar values below 6 cmbsf.

### Relationships between OM content and composition and microbial abundances

**Figure 4:**
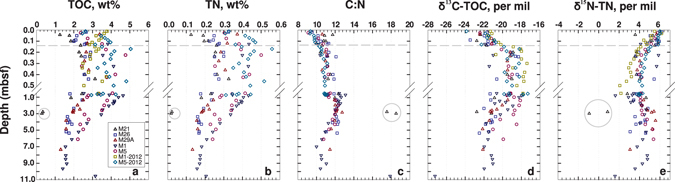
Sediment depth profiles of (**a**) TOC, (**b**) TN, (**c**) C:N, (**d**) δ^13^C-TOC, and (**e**) δ^15^N-TN at 5 stations in Aarhus Bay. Due to problems with the TN measurements on samples from M1-2012, these samples were omitted from (**b**) and (**c**). The horizontal grey dashed lines indicate the minimum depth of macrofaunal sediment reworking (0.14 mbsf).

If BARs in surface sediments are influenced by macrofaunal activity, then macrofaunal influences on the supply and quality of OM are a logical driver of the observed bacterial and archaeal abundance trends, given that the majority of sediment microorganisms are organoheterotrophs. Similarly, the amount and composition of OM below the bioturbation zone would likely have an effect on the abundances of Bacteria and Archaea in deeper non-bioturbated layers. We expanded our OM data set and compared bacterial and archaeal abundances to proxies for OM content (TOC, TN) and OM quality (C:N, δ^13^C-TOC, and δ^15^N-TN) at all Aarhus Bay sites. As already seen for M1-2012 and M5-2012, TOC values in surface sediment are scattered showing no clear relationship with bacterial or archaeal abundances (Fig. 4a; range: 1–5 wt%). Yet, the general decrease in TOC with depth below the bioturbated surface layer (range: ~1.5 to 2.8 wt% below 4 mbsf) falls within the same depth interval over which a gradual decrease in bacterial and archaeal abundances and BARs was observed. The same trend, i.e. scatter with no clear relationship to gene abundances or BARs in surface sediment, and a possible relationship in deeper layers can be seen for TN (Fig. 4b). The glacial till layer at M21 remains an exception, with TOC (0.4 to 0.6 wt%) and TN (~0.02 wt%) values that are lower and far outside the range of all other samples. C:N ratios show an overall increase throughout the top ~1 m of sediment, with the steepest increase in the bioturbated layer, and decrease below (Fig. 4c). The range varies from ~8.8 to 11.6 in surface sediment, to ~10.7 to 13.0 at 1 mbsf, and ~10.0 to 12.3 below 4 mbsf, and is consistent with a predominantly marine origin of the OM. Exceptions are the glacial till layer at M21 and the deep terrestrial soil layer at M1; here, consistent with the terrestrial origin of the OM, C:N values are higher and range from ~17 to 18.

The strongest similarity to BAR trends in surface layers occurs in the δ^13^C-TOC values (Fig. 4d). An increase, from values of ~ −24 to −21‰ to values of ~−21 to −18‰, occurs from surface sediment to sediment immediately underlying the bioturbated sediment layer, indicating a change in OM composition. Below the depth of bioturbation, δ^13^C-TOC values decrease gradually to values that are mostly between −23 and −20‰ below 4 mbsf. Exceptions are the deep terrestrial layers at M21 and M1 (M21: −23.7 to −22.9‰; M1: −27.2‰). The δ^15^N-TN also show a decrease in surface sediments (Fig. 4e); however, this decrease matches the BAR changes to a lesser extent than the δ^13^C-TOC data, as it occurs more steadily throughout the top 50 cmbsf, from ~ +5.3 to +6.2‰ at the sediment surface to +2.0 to +4.2‰ at 50 cmbsf. Below 50 cmbsf, the δ^15^N-TN increases slightly, fluctuating between + 3.0 to 5.3‰ below 4 mbsf. Again, the terrestrial layers are exceptions (M21: −1.0 to +1.8‰, M1: +1.6‰).

### Statistical relationships between 16S gene abundances and geochemical data

To assess the significance of observed trends in BARs in relation to geochemical data, we performed several statistical analyses. Using Kruskal-Wallis tests with Mann-Whitney post-hoc tests, we compared archaeal and bacterial 16S gene abundances and BARs between 4 biogeochemically defined zones (bioturbation zone, sulfate zone, SMTZ, and methane zone). Based on the uniform ^210^Pb_unsupp_ profiles at M1 and M5, which indicate macrofaunal sediment mixing to at least 14 cmbsf, the sediment layer from 0–14 cmbsf was conservatively defined as the bioturbation zone. The sulfate zone extended from below 14 cmbsf to the SMTZ, the SMTZ was the interval in which concentration ratios of sulfate to methane ranged from 2:1 to 1:2, and the methanogenesis zone was the zone below the SMTZ where methane accumulated to millimolar concentrations. As a result of site-specific differences in microbial activity, the depths of the sulfate zone, SMTZ and methane zones vary between stations (Supplementary Table 1, Supplementary Fig. 2). Kruskal-Wallis tests revealed that mean archaeal 16S gene abundances do not differ significantly between any of the biogeochemical zones, except between the sulfate zone and the methane zone (Table 1). By contrast, mean bacterial 16S gene abundances, and BARs are significantly higher in bioturbated sediments than in the other biogeochemical zones. Mean bacterial 16S gene abundances and BARs are also significantly higher in the sulfate zone than in the SMTZ or methane zone, but do not differ significantly between the SMTZ and methane zone.

**Table 1:** Comparison of bacterial and archaeal 16S gene abundances and BARs across four biogeochemical zones based on Kruskal-Wallis tests with Mann-Whitney post-hoc tests.

Archaea				
	Bioturbation zone	Sulfate zone	SMTZ	Methane zone
Bioturbation zone	/	0.30	0.38	0.13
Sulfate zone	0.30	/	0.17	**<0.05**
SMTZ	0.38	0.17	/	0.69
Methane zone	0.13	**<0.05**	0.69	/
**Bacteria**				
Bioturbation zone	/	**<0.01**	**<0.01**	**<0.01**
Sulfate zone	**<0.01**	/	**<0.05**	**<0.01**
SMTZ	**<0.01**	**<0.05**	/	0.38
Methane zone	**<0.01**	**<0.01**	0.38	/
**BAR**				
Bioturbation zone	/	**<0.01**	**<0.01**	**<0.01**
Sulfate zone	**<0.01**	**/**	**<0.01**	**<0.005**
SMTZ	**<0.01**	**<0.01**	/	**0.09**
Methane zone	**<0.01**	**<0.005**	0.09	/

Statistically significant p-values are shown in bold. Only stations from which all 4 biogeochemical zones were reached by coring were included (M24, M26, M27A, M29A, M1 (2009), and M5-2012).

We furthermore performed correlation analyses between microbial abundance and TOC, TN, C:N, δ^13^C-TOC, and δ^15^N-TN data (Supplementary Fig. 5). Because TOC, TN, and δ^13^C-TOC displayed trends in relation to the depth of bioturbation (Fig. 4), we subdivided sediments into bioturbated (0–14 cmbsf) and non-bioturbated (below 14 cmbsf). We then used Spearman Rank Correlation tests and effect size calculations (coefficient of determination (R^2^)), based on deviations from best-fit lines, to explore these relationships further. In surface sediment, significant correlations were observed for BARs, archaeal and bacterial gene copies in relation to δ^13^C-TOC (Supplementary Fig. 5a). BARs and bacterial gene copies decreased significantly with increasing δ^13^C-TOC (p < 0.001), whereas archaeal gene copies increased significantly with increasing δ^13^C-TOC (p < 0.05). BARs and bacterial gene copies displayed a better fit to a trend line, with R^2^ values of 0.51 and 0.43, compared to archaeal gene copy numbers, which were more scattered (R^2^ = 0.18). No significant relationships between BARs, bacterial and archaeal gene copy numbers and TOC, TN, or C:N were observed. No significant correlations with δ^15^N-TN were observed except for bacterial gene copies (p < 0.05); yet, the effect size of this relationship was very low (R^2^ = 0.03).

In the underlying non-bioturbated sediments, different patterns were found. BARs, archaeal and bacterial gene copy numbers were significantly correlated with TOC, TN, C:N, and δ^13^C-TOC (Supplementary Fig. 5b). BARs, but not archaeal or bacterial gene copy numbers, were also significantly correlated with δ^15^N-TN. Effect sizes for relationships between archaeal and bacterial gene copy numbers and TN were the largest (R^2^: 0.49 and 0.59, respectively), followed by relationships between archaeal and bacterial gene copy numbers and TOC (0.40 and 0.44, respectively), and the relationship between bacterial gene copy numbers and C:N (0.35). All other relationships, including ones between BARs and OM variables, were characterized by stronger scatter and lower R^2^ values. These trends are independent of whether the terrestrial samples are included. Compared to Supplementary Fig. 5b, we observe similar trends and R^2^ values in Supplementary Fig. 5c, where only samples from Holocene mud layers are included.

### Method checks

Due to well-documented DNA extraction and PCR biases, which may affect the results of PCR-based quantifications of microorganisms, we performed additional method checks (for details see ‘Method comparisons’ in Supplementary Results). We compared bacterial and archaeal 16S gene abundance data to the same data obtained with a widely used commercial DNA extraction kit and using two additional bacterial and two additional archaeal 16S gene primer combinations. Independent of the DNA extraction method and primer choice, the overall trends in bacterial and archaeal 16S genes were confirmed (ref. 14, Fig. 10c,d; Supplementary Fig. 6). We also compared total 16S gene copy numbers to cell count data, to determine whether 16S gene copy numbers were reproducible using a different microbial quantification method. Total 16S gene abundance trends showed good agreement with the cell count data (Supplementary Fig. 8).

## Discussion

Our results are in line with previous studies showing bacterial dominance in surface sediments^22, 24–26^ and similar population sizes of Archaea and Bacteria in subsurface sediments^7, 9, 12^ (Fig. 1). Yet, our results for the first time indicate that the dominance of Bacteria over Archaea in surface sediment might be the outcome of benthic macrofaunal activity. BARs range from 10 to 60 in the top sediment layer (0–6 cmbsf), where the absence of depth gradients in ^210^Pb_unsupp_, δ^13^C-TOC, DIC and sulfate concentrations indicate high rates of macrofaunal sediment reworking and bioirrigation (Figs 2 and 3). Below (~6–14 cmbsf), as macrofaunal activity gradually decreases with depth, bacterial 16S gene abundances decrease by one order of magnitude, whereas archaeal 16S gene abundances increase (Figs 1 and 2, Supplementary Fig. 2). A clear increase in δ^13^C-TOC over the bioturbated interval (Fig. 4d) suggests a shift in OM composition from the highly bioturbated surface layer to deeper non-bioturbated sediments. Further below (14–20 cmbsf), macrofaunal particle mixing and irrigation cease, and relative abundances of archaeal and bacterial 16S genes approach an average ratio of 2:1, a ratio that remains stable throughout the sulfate zone and decreases to circa 1:1 in the SMTZ and methane zone (Supplementary Fig. 2, Supplementary Table 2). In addition to the biogeochemical zone, TOC and TN data suggest that OM content is a main driver of archaeal and bacterial abundances and BARs below the depth of bioturbation (Fig. 4a,b, Supplementary Fig. 4b).

The observed trends in archaeal and bacterial abundances are replicated across all 8 stations in Aarhus Bay, but are less evident at a non-bioturbated control site in Aarhus Harbor. The dominance of bacterial 16S genes in bioturbated sediment is, moreover, independent of primer choice (Supplementary Fig. 6) and reproducible with a commercial DNA extraction kit^14^. Comparisons between total abundances of 16S genes and total cell abundances from four stations indicate that 16S gene copy numbers per cell are between 1–5 in most samples, with site-specific averages ranging from 2.0 to 4.3 (Supplementary Fig. 8b). These values are typical of microbial populations in other environments^35, 36^ and indicate good agreement between two independent quantification methods.

The mechanisms by which sediment macrofauna might affect archaeal and bacterial gene abundances in surface sediments are diverse. Valentine^27^ proposed that Archaea are better adapted to life under chronic energy limitation than Bacteria, and that Bacteria on the other hand are better adapted to dynamic and variable environments where energy limitation is not permanent. These notions may help explain why macrofaunal activity selects for Bacteria-dominated communities in surface sediments. Macrofaunal reworking strongly increases microbial energy availability by introducing labile OM from overlying water and the sediment surface to deeper layers, and by converting large organic particles into more microbially accessible small organic particles and dissolved OM^28, 37, 38^. Bioirrigation introduces high-energy electron acceptors, e.g. O_2_ and nitrate, which increase rates of OM degradation and promote chemical and chemolithotrophic reoxidation of reduced metals, sulfide, and ammonium produced by microbial respiration^39–41^. Since both reworking and bioirrigation are spatially and temporally variable processes, bioturbation inevitably converts surface sediments into a heterogeneous and dynamic environment where a stable redox zonation is absent. Translated to patterns we observe in Aarhus Bay, macrofauna may favor dominance of Bacteria in bioturbated surface sediments by enhancing the input and availability of both OM and high-energy electron acceptors, and by increasing physiological stress through redox fluctuations. Archaea may in turn increase relative to Bacteria in deeper sediments with little to no bioturbation due to chronic energy limitation, resulting from the predominance of recalcitrant OM and low-energy electron acceptors, such as sulfate and CO_2_, or due to reduced physiological stress resulting from a more stable redox potential.

The notion of enhanced energy availability due to macrofaunal input and breakdown of OM in surface sediment is consistent with our OM data and with previous studies indicating a decrease in cell-specific sulfate reduction rates below 10 cm at M1^32, 42^. Though our TOC, TN, or C:N data show no clear trend in relation to BARs, our δ^13^C-TOC profiles show a clear shift from bioturbated surface to non-bioturbated subsurface sediment that matches the depth and rates of sediment reworking based on ^210^Pb_unsupp_ (Fig. 4). δ^13^C-TOC values of −24 to −21‰ in surface sediment (Figs 2i,j and 4d) are consistent with a mainly phytoplankton origin of OM^43^, whereas the shift to values of −20 to −18‰ in deeper, non-bioturbated layers indicates an increased contribution of a different OM pool with higher δ^13^C-TOC values. This OM pool likely includes seagrass, which has a typical δ^13^C-TOC range of −15 to −3^39^, is resistant to microbial degradation^44^ consisting largely of recalcitrant polymers such as cellulose, hemicellulose, lignin, and cutin^45^, and is widely found in Aarhus Bay (*Zostera marina*)^46^. Selective feeding and mineralization of labile, more rapidly biodegradable phytoplankton-derived OM over more recalcitrant OM from seagrass would explain the observed increase in δ^13^C-TOC from the sediment surface downward through the bioturbation zone. Bacterial dominance in bioturbated sediments would then be promoted by higher growth rates of Bacteria compared to Archaea in the presence of labile OM.

Support for an important role of bioirrigation in Aarhus Bay surface sediment comes from past measurements of the depth distributions of aerobic and anaerobic respiration reactions at M1^34^, model calculations which suggest that >90% of sulfate in sediment of M1 and M5 is introduced by bioirrigation^33^, and from our DIC and sulfate data. In the SML, where BARs are the highest (Figs 1 and 2), bioirrigation activity is the highest based on sulfate and DIC concentrations that are close to those in overlying seawater (Fig. 2g,h). Below the SML, the slopes of the sulfate concentration profiles and the δ^13^C-DIC profiles indicate reduced but significant introduction of seawater by bioirrigation down to ~20 cmbsf (Fig. 2g–j). If bioirrigation is the main driver of the observed distributions in bacterial and archaeal gene abundances in surface sediments, then the higher BARs in bioirrigated sediments could result from increased energy availability due to import and regeneration of high-energy electron acceptors or enhanced physiological stress due to redox fluctuations caused by fluctuating concentrations of these same high-energy electron acceptors.

If enhanced energy input and/or redox fluctuations due to macrofauna explain the dominance of Bacteria over Archaea within the bioturbated surface layer, then what controls the abundances of Bacteria and Archaea in sediments underlying the bioturbation zone? Our data indicate that two factors are important: the biogeochemical zone, i.e. sulfate zone, SMTZ, or methanogenesis zone, and the total amount of OM. Given that sulfate-dependent anaerobic oxidation of methane (AOM) is a quantitatively important process in SMTZs, that is only performed by Archaea, and that methanogenesis is the dominant respiration pathway in sulfate-depleted sediments of the methane zone, and also only performed by Archaea, it is not surprising that BARs are higher in sulfate zones, where AOM and methanogenesis are less important, compared to SMTZs or methane zones. More interesting is, perhaps, that the quantitative importance of OM content (TOC, TN) in controlling archaeal and bacterial abundances in subsurface sediments appears to exceed the quantitative importance of OM composition (C:N, δ^13^C-TOC) (Supplementary Fig. 4b). A possible explanation is that energy availability, and thus energy substrate availability, to microorganisms in subsurface sediments is directly related to OM content. Accordingly, differences in OM lability/reactivity or nutrient content may be less important in subsurface layers, because the bulk of OM is highly recalcitrant, having been selectively stripped of readily bioavailable and nutrient-rich OM fractions by diagenetic processing while passing through shallow layers.

**Figure 5:**
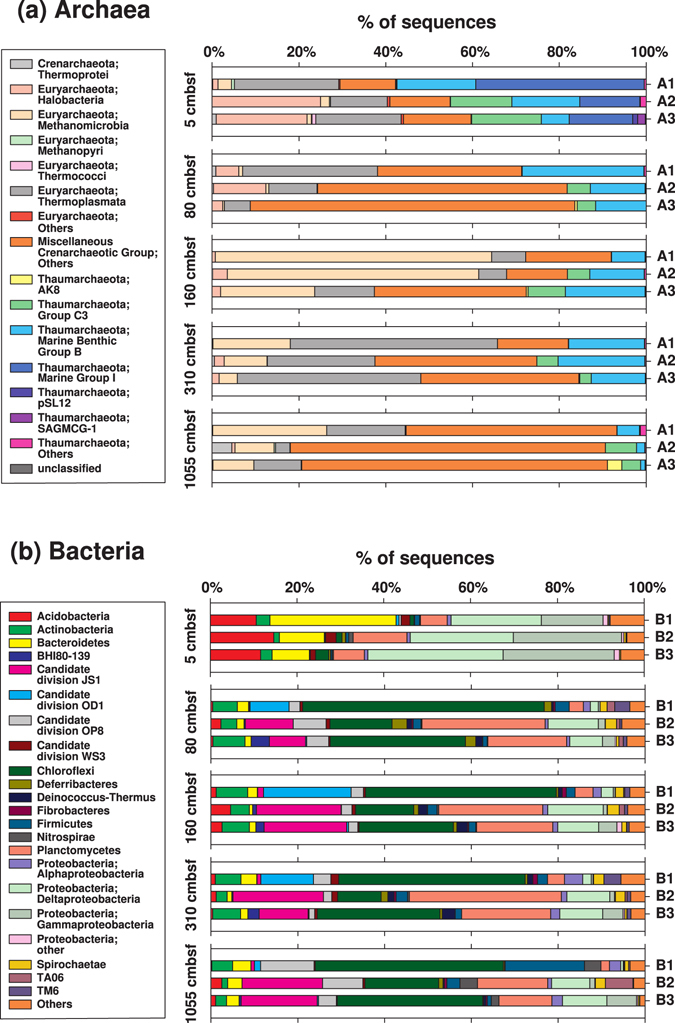
Archaeal and bacterial community compositions in the bioturbation zone (5 cmbsf), the sulfate zone (80 cmbsf), the SMTZ (160 cmbsf), the shallow methane zone (310 cmbsf), and in a freshwater peat layer within the deep methane zone (1055 cmbsf) at M1. Archaeal 16S gene libraries were produced using the primer pairs A1 (Arc806F - Arc958R), A2 (Arc806F - Arc915Rmod), and A3 (Arc915Fmod - Arc1059R), while the bacterial libraries were produced using the primer pairs B1 (Bac8F - Bac338Rabc), B2 (Bac806F_mod2 - Bac908R), and B3 (Bac908F - Bac1075R). Operational Taxonomic Units (OTUs; 97% cutoff) were assigned to class level for Archaea and to phylum level for Bacteria using Mothur, based on an alignment of amplicons to the 16S rRNA reference sequences in SILVA Release 119. See Supplementary Methods for more information.

While the use of qPCR on archaeal and bacterial 16S genes provides an efficient means to identify depth trends in abundances of the domains Archaea and Bacteria, insights into possible changes in archaeal and bacterial community structure are not obtained. Using the same archaeal and bacterial primer combinations as in the qPCR primer comparison (for details see ‘Additional Primer Checks’ in Supplementary Methods and Supplementary Fig. 7), we therefore sequenced 16S genes of Archaea and Bacteria from 5 depth horizons at M1. Independent of primer pair, we observe a clear shift in archaeal and bacterial community composition from bioturbated to non-bioturbated sediment (Fig. 5, Supplementary Fig. 8). For Archaea, the main vertical changes in community composition are the disappearance of Marine Group I Thaumarchaeota from bioturbated sediment to underlying horizons (Fig. 5a). Furthermore, Methanomicrobia, which account for a small fraction of reads in the bioturbation and sulfate zones, become more prominent in the SMTZ and methane zone. Neither of these changes is surprising. Marine Group I Thaumarchaeota consist of aerobic ammonia- and urea-oxidizing Archaea that are widespread in marine water columns^47^ and presumably extend their range into surface sediments thanks to bioirrigating macrofauna which regularly import O_2_ into these layers. Methanomicrobia consist of methanogenic and anaerobic methane-oxidizing Archaea. Methanogens are mainly found in sediments where respiration reactions involving O_2_, nitrate, metal oxides, or sulfate, which have higher energy yields than methanogenesis, are absent^48^, whereas anaerobic methane oxidation occurs mainly in SMTZs in marine sediments^49^. The vertical changes in dominant bacterial groups are even more dramatic than those in Archaea (Fig. 5b). Acidobacteria, Bacteroidetes, Deltaproteobacteria, and Gammaproteobacteria account for ~80% of sequence reads in the bioturbated layer, and for ≤20% of sequence reads in underlying horizons. By contrast, in the four subsurface horizons, the bacterial community consists to ~70% of Chloroflexi, Planctomycetes, Candidate Division JS1 (Atribacteria), Candidate Division OD1, and Candidate Division OP8. Though a division of these groups into functional guilds is confounded by the metabolic diversity within certain groups, e.g. Delta- and Gammaproteobacteria, and limited knowledge on others, e.g. Chloroflexi, Bacteroidetes, and the Candidate divisions, the clear shift in bacterial community composition from bioturbated surface sediments to underlying horizons that are cut off from fresh OM and high-energy electron acceptor inputs by macrofauna is apparent.

Interestingly, despite the observed changes in BARs from the sulfate zone to the methane zone at M1, the fraction of typical marine sulfate reducing Bacteria, such as Deltaproteobacteria and Firmicutes, is small in non-bioturbated sediment of the sulfate zone (~10%) and does not change from the sulfate to the methane zone. Furthermore, methanogens also only account for a small fraction of archaeal sequence reads in the methane zone (~5–25%). This trend of sulfate reducers and methanogens accounting for only minor fractions of the total microbial community in sulfate reducing and methanogenic subsurface sediments has been documented previously^50^. It suggests that the change in BARs from the sulfate zone to the methane zone at M1, and possibly other stations in Aarhus Bay, is not solely driven by population trends among sulfate reducers and methanogens, but that the population sizes of other groups of Bacteria and Archaea are changing as well.

By quantifying archaeal and bacterial 16S gene abundances, we are ultimately aiming to quantify relative abundances of Archaea and Bacteria. This approach has several caveats, however. Mean ± Standard deviations of 16S gene copies per cell differ between these domains, with mean bacterial (average ± SD: 4.12 ± 2.75 copies cell^−1^) exceeding mean archaeal values (average ± SD: 1.61 ± 0.88 copies cell^−1^) by a factor of 2.5 (https://rrndb.umms.med.umich.edu/). This results in uncertainties whenever bacterial and archaeal cell abundances are estimated by quantifications of 16S genes. For instance, in the sulfate zone and SMTZ, where bacterial 16S genes are on average twice as abundant as archaeal 16S genes, actual abundances of bacterial and archaeal cells may be in the same range. In the methane zone, where 16S gene abundances of Archaea and Bacteria are roughly equal, archaeal cells may even outnumber bacterial cells. Moreover, if bacterial groups with high average 16S gene copy numbers per cell, such as Firmicutes (average ± SD: 6.46 ± 3.02 copies cell^−1^) and Gammaproteobacteria (average ± SD: 5.66 ± 2.47 copies cell^−1^) happened to dominate bioturbated sediments, then actual cell-to-cell ratios of Bacteria to Archaea in surface sediments might be much lower than the 16S gene ratios. Yet, based on our already discussed sequence data (Fig. 5), which was obtained with three different bacterial primer combinations, this alternative scenario is unlikely. Firmicutes and Gammaproteobacteria account for <1 and 15–25% of bacterial 16S gene sequences in the bioturbated layer, respectively, and are outnumbered there by bacterial groups that typically have low 16S gene copy numbers per cell, such as Acidobacteria (average ± SD: 1.38 ± 0.48 copies cell^−1^), Bacteroidetes (average ± SD: 3.21 ± 1.74 copies cell^−1^), and Deltaproteobacteria (average ± SD: 2.70 ± 1.33 copies cell^−1^); these latter groups account for 10–15%, 8–30% and 20–30% of bacterial 16S gene sequences in the bioturbated layer, respectively. Furthermore, if 16S gene copy numbers were substantially higher in Bacteria inhabiting bioturbated surface sediments compared to subsurface sediments, then our calculated 16S genes per cell at M1 should clearly decrease with depth; however, we see no decrease in mean 16S genes per cell with sediment depth (Supplementary Fig. 8b). Consequently, our data indicate that the vastly higher bacterial than archaeal 16S gene abundances reflect a strong numerical dominance of Bacteria over Archaea in the bioturbated layer and are not the result of higher bacterial 16S gene copy numbers per cell.

In conclusion, our study indicates that bioturbation is a key driver of microbial community structure in marine surface sediments. Previous studies have documented shifts in relative abundances of Archaea and Bacteria similar to the ones we observe in surface sediments, but not linked these changes to bioturbation^24–26^. Other studies have documented differences in microbial biomass and community structure between burrow walls and surrounding sediment^51–53^. Our data suggest that bioturbation is a key driver behind the observed shifts in BARs in surface sediments, and strongly influences microbial community structure not only in burrow walls but throughout the entire bioturbated sediment interval. The observed changes in BARs in Aarhus Bay match the typical depth interval of macrofaunal activity in marine sediments^54^. Given that it is a globally important process that dominates particle transport in marine surface sediments^55^ and occurs from organic-rich coastal environments to oligotrophic ocean gyres^56^, bioturbation could be a widely overlooked driver of microbial community structure in marine surface sediments worldwide. The strong shifts in BARs from the bioturbated surface layer to the underlying non-bioturbated layers suggest that the lower parts of bioturbation zones represent an important ecological transition, in which surface sediment microbial communities change to phylogenetically and physiologically distinct subsurface communities. Future studies involving closely monitored manipulation experiments will reveal how macrofaunal influences on OM content, electron acceptor availability, and redox fluctuations each affect microbial community structure, and how the influence of bioturbation on microbial community structure depends on macrofaunal species composition, macrofaunal feeding mode, and the broader environmental setting.

## Methods

### Sediment sampling

All stations in central Aarhus Bay were sampled by gravity corer and by Rumohr Lot corer^57^ in October 2009 and May 2010 (Supplementary Table 1). Use of gravity corers enabled recovery of sediments down to depths of 3–11 m. Use of 1-m Rumohr corers enabled the recovery and high-resolution sampling of undisturbed surface sediments to depths of 0.5–0.8 m. Sediment sampling for analyses of archaeal and bacterial 16S gene abundances was done every 8 cm in Rumohr cores and every 10–20 cm in gravity cores.

Stations M1 and M5 were revisited in March 2012 for additional sampling with a Rumohr corer to study relationships between archaeal and bacterial 16S gene abundances and macrofaunal bioturbation in surface sediments at high depth resolution. The sampling resolution was every 2 cm in the upper 20 cm, every 4 cm from 20–40 cm, and every 8 cm below. Additionally, a 15-cm push core was taken in Aarhus Harbor in June 2012 and sectioned at 2-cm depth intervals.

### Site descriptions

Aarhus Bay is a shallow semi-enclosed embayment on the transition between the North Sea and the Baltic Sea, characterized by 6–7 m thick methane-rich Holocene mud overlying late glacial clay-silt and till and early Holocene peat^31, 58^. According to ^14^C-dating of marine bivalve and gastropod shells, the postglacial marine transgression which led to fully marine conditions started in Aarhus Bay about 8,500 years ago^31, 59^. Most of the anaerobic microbial mineralization of OM is coupled to the reduction of sulfate and only a few percent to the production of methane^32^. Sulfate reduction rates are highest in the uppermost anoxic sediment and show a subsurface peak in the SMTZ^33, 60^.

The eight stations of this study are from the central basin of Aarhus Bay (Supplementary Fig. 1): M21 and M22A have no significant methane accumulation in the cored interval. M24 and M26 have shallow methane production but no free CH_4_ gas in the cored interval, and M27A, M29A, M1, and M5 have shallow methane production and free CH_4_ gas at variable depths within the cored interval (Supplementary Table 1). Sediments at all stations consist of organic-rich mud with two exceptions: M21 has organic-poor glacial sand below ~2 m sediment depth, whereas M1 has early Holocene peat below 10.5 m sediment depth. With the exception of M21, depth profiles of pore water concentrations of sulfate, methane, dissolved inorganic carbon (DIC) and other ions, TOC, total organic nitrogen (TON), δ^13^C-TOC, δ^15^N-TN, and C:N, and rates of anaerobic mineralization of OM have been published (M22A-M29A^32^, M1^60, 61^, M5^33, 62^).

All eight Aarhus Bay stations show visual evidence of bioturbation based on sediment color, and presence of burrows and macrofauna. These observations are consistent with sulfur, iron, and manganese mass balance calculations and ^210^Pb_unsupp_ distributions indicating that surface sediments are geochemically influenced by bioturbation that maintains oxidizing, yet anoxic conditions^34^. Macrofaunal densities are high (2800 ± 700 individuals/m^2^), and the benthic fauna is dominated by bivalves (*Abra alba*, *Mysella bidendata*, *Corbula gibba*, *Macoma calcarea*), polychaetes (e.g., *Terebellides stroemi*, *Nephtys ciliata*, *Nephtys sp*., *Heteromastus filiformis*, *Pectinaria sp*.), and echinoderms (e.g., *Ophiura albida*, *Echinocardium cordatum*)^34, 63^. An overview of the size, burial depth and feeding mode of these macrofauna is shown in Supplementary Table 3. Surface deposit and suspension feeding bivalves (mainly *A*. *alba*) are by far the most abundant group, accounting for ~50–90% of macrofaunal individuals and ~40–≥95% of total macrofaunal biomass^64^. Polychaetes are the second most abundant group, accounting for ~5–30% of all individuals and >1–60% of total biomass, and are dominated by the genus *Nephtys*. A site in Aarhus Harbor (AHB) served as a bioturbation control site due to the absence of visible bioturbation.

Detailed descriptions of the Rumohr cores taken in March 2012 from M1 and M5, and the push core from AHB are provided in the Supplementary Methods.

### Sediment DNA extraction

Samples for DNA extraction were taken by sterile, cut-off syringes and stored at −80 °C. DNA was extracted according to Lever *et al*.^14^ using a protocol that had been developed during extensive extraction tests with Aarhus Bay sediment. After initial removal of sDNA, i.e. dissolved DNA and DNA that was likely adsorbed to sedimentary matrices and became dissolved after soaking in an alkaline phosphate buffer, cells were chemically lysed via lysis protocol III, washed with chloroform-isoamylalcohol, precipitated by ethanol-NaCl solution with linear polyacrylamide (LPA) as a co-precipitant (20 µg LPA mL^−1^ extract), and purified using the CleanAll RNA/DNA Clean-Up and Concentration Kit (Norgen Biotek Corp., Canada; for details see ref. 14).

### Quantification of archaeal and bacterial 16S genes

Abundances of archaeal and bacterial 16S genes were determined on a Roche LightCycler 480. Archaeal genes were quantified using the primer sets Arch 806F^65^ (*ATT AGA TAC CCS BGT AGT CC*) and Arch 958R^66^ (*YCC GGC GTT GAM TCC AAT*), while bacterial genes were quantified using 8Fmod^67^ (*AGA GTT TGA TYM TGG CTC AG*) and 338Rabc^68^ (*GCW GCC WCC CGT AGG WGT*). Reaction volumes of 20 µL consisted of 1 × Light Cycler Mastermix (Roche), 1 µg µL^−1^ bovine serum albumin (BSA), 0.5 µM of each primer, 2 µL template, and molecular-grade water. Plasmids of 16S genes from *Methanosarcina*-affiliated archaea and *Sphingomonadales*-affiliated bacteria were used as standards. Standard series covered gene concentrations of 10^1^–10^8^ copies µL^−1^. The thermal cycler protocol consisted of (1) enzyme activation and initial denaturation at 95 °C for 5 min; (2) 45 cycles of (a) denaturation at 95 °C for 30 s, (b) annealing at 55 °C for 30 s, (c) elongation at 72 °C for 10 s (Archaea) or 15 s (Bacteria), and (d) fluorescence measurement at 78 °C (Archaea) or 80 °C (Bacteria) for 15 s; and (3) a stepwise melting curve from 95 C to 55 C in 1 min to check for primer specificity. All standards and samples were analyzed in triplicate. To check for PCR inhibition, we compared gene quantifications of original extracts to 1:10 dilutions. Due to slight PCR inhibition in original extracts, values of 1:10 dilutions were used to estimate the abundances of 16S genes in samples.

### Microbial cell counts

Microbial populations at M22A, M27A, M29A, M1, and M5 were also quantified by cell counts on cores taken in May 2010. Cells were extracted using a protocol optimized for Aarhus Bay sediment^62^, stained with Sybr Green I, and enumerated by epifluorescence microscopy with an automated stage and slide-loader combined with an image analysis software program^69^. Cell abundances were then compared to the sum of archaeal and bacterial 16S genes measured by qPCR. Whenever cell abundances were not available from the same depth as qPCR data, data from the closest available depths were compared (depth difference: <4 cm for samples above 1 mbsf, and <13 cm for samples below 1 mbsf).

### Geochemical analyses

Methods for the sampling and quantification of sulfate, methane, DIC, and δ^13^C-DIC in cores taken in October 2009 and May 2010 are described in ref. 32. Two Rumohr Lot cores were sampled at each station, one for methane quantification, DNA extraction, and cell counts, the other for sulfate, DIC, and δ^13^C-DIC. One gravity core was taken at each station, which was used for all of the above geochemical analyses, DNA extractions, and cell counts.

Sulfate, DIC, and δ^13^C-DIC measurements on samples obtained from M1 and M5 in March 2012 and the Aarhus Harbor reference site were done in the same way, except that porewater samples were obtained by centrifugation rather than by rhizons and processed as follows. For sulfate measurements, 1.5-mL Eppendorf tubes were filled with sediment and centrifuged for 10 min at 10,000 × g and 4 °C. The supernatant was transferred and immediately acidified and stripped of hydrogen sulfide by gentle bubbling with CO_2_ (*g*) for 1 min, after which samples were frozen at −20 °C until analysis. DIC samples were obtained by filling up 50-mL Falcon tubes with sediment, centrifugation for 10 min at 5,000 × *g*, followed by filtration of supernatant through 0.22-µm pore size polyethersulfone membrane filters (Cronus biotech Ltd.) into 2-mL borosilicate vials that were filled completely and stored at +4 °C until analysis. Methane sampling and analyses on March 2012 cores was done as explained in ref. 32.

TOC, TN, δ^13^C-TOC, and δ^15^N-TN were sampled and analyzed as described previously^62^. Samples were always taken in direct vicinity to samples used for DNA extractions within the same Rumohr Lot or gravity core.

### Radioisotope measurements and modeling

^210^Pb (half-life: 22.3 years), ^226^Ra (1,600 yrs), and ^137^Cs (30.2 years) radioactivity were measured on Rumohr cores taken at M1 and M5 in March 2012 and analyzed via gamma-spectrometry. 2-cm depth intervals were pooled and dried as described for TOC^62^. Measurements were carried out on a Canberra ultralow-background Ge-detector. ^210^Pb was measured via its gamma-peak at 46.5 keV, ^226^Ra via the granddaughter ^214^Pb (peaks at 295 and 352 keV), and ^137^Cs via its peak at 661 keV. ^210^Pb_unsupp_ was calculated by subtracting supported ^210^Pb values based on ^226^Ra decay from measured ^210^Pb values. Long-term (>1000 years) sedimentation rates at both stations were determined by ^14^C (half-life: 5,730 years) analyses on bivalve shells (Rasmussen *et al*., *unpubl*.) and indicate net erosion at M1, where surface sediments have an extrapolated age of 2,800 yrs, and a net sedimentation rate of 1 mm yr^−1^ at M5. The mixing coefficient, D_b_, was calculated using the following equation from Huh and Su^70^, assuming sedimentation rates of 0 mm yr^−1^ at M1 and 1 mm yr^−1^ at M5 and a constant D_b_ in the layer, in which the concentration of ^210^Pb_unsupp_ decreases exponentially with depth

$${\rm{C}}={{\rm{C}}}_{0}\,\exp ((({\rm{S}}-{({{\rm{S}}}^{2}+4\lambda {{\rm{D}}}_{{\rm{b}}})}^{0.5})/2{{\rm{D}}}_{{\rm{b}}})\,{\rm{Z}})$$

where C = activity of the radionuclide (Bq kg^−1^ dwt) at depth z (cm), C_0_ = activity at the surface, λ = radioactive decay constant (0.0311 yr^−1^), D_b_ = mixing coefficient (cm^2^ yr^−1^), S = sedimentation rate (cm yr^−1^). For a review on the use of radionuclides to measure rates of macrofaunal sediment reworking, we recommend Maire *et al*.^71^ and the references within.

### Statistical analyses

To determine whether archaeal and bacterial 16S genes, BARs, and cell abundances differed significantly between the four biogeochemical zones, we performed Kruskal-Wallis tests with Mann-Whitney post-hoc tests using the software PAST^72^ (http://folk.uio.no/ohammer/past/). We used station-specific averages for each biogeochemical zone (Supplementary Table 2) in our calculations. All trendline analyses and calculations of R^2^ were done in Microsoft Excel. Spearman Rank Correlation tests were performed using the free, open-source online statistical package Wessa.net (http://www.wessa.net/)^73^.

### Archaeal and bacterial community compositions at M1

To examine the archaeal and bacterial community composition change at the four biogeochemical zones, we sequenced the 16S gene PCR products generated by three archaeal and three bacterial primer combinations from five depths at M1 (5, 80, 160, 310 and 1,055 cmbsf) using an Ion Personal Genome Machine (PGM™) system (Ion Torrent, Life Technologies). For further details, see the Supplementary Methods.

## Electronic supplementary material

Supplementary Information
