# Supplementary material for: Bioturbation as a key driver behind the dominance of Bacteria over Archaea in near-surface sediment
Source: Sci Rep. 2017 May 25;7:2400. doi: 10.1038/s41598-017-02295-x (PMC5445093; doi:10.1038/s41598-017-02295-x)
Supplement: Supplementary file 1 — Supplementary Information [file 41598_2017_2295_MOESM1_ESM.pdf]

SUPPLEMENTARY INFORMATION

Bioturbation as a key driver behind the dominance of Bacteria over Archaea in near-surface sediment

Authors: Xihan Chen [1], Thorbjørn Joest Andersen [2], Yuki Morono[3], Fumio Inagaki[3], Bo Barker Jørgensen [1], Mark Alexander Lever* [1,4]

Author affiliation:

[1] Center for Geomicrobiology, Department of Bioscience, Aarhus University, 8000 Aarhus, Denmark

[2] Department of Geosciences and Natural Resource Management, University of Copenhagen, 1350 Copenhagen, Denmark

[3] Kochi Institute for Core Sample Research, Japan Agency for Marine-Earth Science and Technology (JAMSTEC), Nankoku, Kochi 783-8502, Japan

[4] Institute for Biogeochemistry and Pollutant Dynamics, Department of Environmental Systems Sciences, ETH Zürich, 8092 Zürich, Switzerland

Correspondence: mark.lever@usys.ethz.ch

Keywords: 16S rRNA gene / Bacteria-to-Archaea Ratio / bioturbation / microbial community /quantitative PCR

Supplementary Results

Method comparisons

Our interpretations of archaeal and bacterial 16S gene abundance profiles assume that the DNA extraction and 16S gene amplification data reflect real trends within sediments rather than biases introduced by the DNA extraction method or primer choices. Using M1 sediment, we had previously shown good comparability in overall trends of archaeal and bacterial 16S gene abundances between our extraction method and a commercial DNA extraction kit (reference #6, Fig. 10c,d). We here additionally performed epifluorescence microscopic cell counts at M22A, M27A, M29A, M1, and M5 (Supplementary Fig. 7) and calculated gene copy numbers per cell by dividing the sum of archaeal and bacterial 16S gene abundances per sample by cell abundance data from the nearest sample in which cell numbers were quantified. These calculations enabled us to check whether calculated average gene copies per cell were in a realistic range. Published global mean±standard deviations of 16S gene copies per cell are 4.12±2.75 for Bacteria and 1.61±0.88 for Archaea (https://rrndb.umms.med.umich.edu/). Average values of 1-7 16S gene copies per cell would thus indicate good agreement between qPCR and cell count data. By contrast, values >7 16S gene copies per cell would suggest poor agreement, e.g. due to inclusion of high numbers of 16S genes from outside of living cells or poor cell extraction and cell enumeration efficiency. Similarly, values of <1 16S gene copies per cell would indicate poor agreement, e.g. caused by low extraction efficiency of the DNA extraction method.

The cell abundance profiles match a previously proposed cell distribution model for marine sediments[29] (Supplementary Fig. 8a). Calculated 16S gene copies per cell mostly range between 1 and 5, suggesting a good match between 16S gene abundances and cell counts (Supplementary Fig. 8b; site averages±standard deviation: M22A: 4.3±4.2; M27: 2.2±1.4; M29A: 4.2±2.5; M5: 2.0±1.4 copies $cell^{-1}$). This good agreement is remarkable

given that cell and 16S gene abundances were quantified using independent extraction and quantification methods, and that DNA was extracted from different sediment aliquots and slightly different sediment depths than cells for cell counts. Noteworthy is also the absence of a clear, depth-related change in calculated 16S genes per cell, in spite of the shift from bacterial dominance in the bioturbation zone to similar population sizes of Bacteria and Archaea in deeper layers. This suggests that Bacteria and Archaea have similar numbers of 16S genes per cell in surface and subsurface marine sediments of Aarhus Bay (also see Discussion).

We furthermore investigated the possibility that our domain-specific qPCR primer pairs introduced strong biases, which led to the observed trends in archaeal and bacterial distributions (info on primer combinations in Supplementary Methods). Indeed, while agreement was good in bioturbated surface sediments, we observed significant, primer-dependent differences in archaeal 16S gene abundance estimates in subsurface sediments, with our archaeal primer choice resulting in higher estimates than two other archaeal primer combinations (Supplementary Fig. 6a). By contrast, bacterial 16S gene abundances estimated using three different bacterial primer pairs showed a better agreement (Supplementary Fig. 6b). Despite these primer-related differences, the overwhelming dominance of bacterial over archaeal 16S genes in surface sediments, and the comparable 16S gene abundances of the two domains in subsurface sediments were confirmed (Supplementary Fig. 6c).

Supplementary Methods

Additional Primer Checks

We performed additional qPCR assays to compare gene copy numbers obtained with three different archaeal and three different bacterial qPCR primer combinations on five sediment

depths from station M1 (5, 80, 160, 310 and 1055 cmbsf). At each sediment depth we performed three replicate DNA extractions. In addition, we ran three replicate qPCR assays on each individual extract. The three archaeal primer pairs used were: (1) Arc806F-Arc958R (described in the Methods), (2) Arc806F-Arc915Rmod (5'-GTG CTC CCC CGC CAA TT-3')[3], and (3) Arc915Fmod (5'-AAT TGG CGG GGG AGC AC -3')[3] - Arc1059R (5'-GCC ATG CAC CWC CTC T-3')[4]. The three bacterial primer pairs were: (1) Bac8Fmod-Bac338Rabc (described in the Methods), (2) Bac806F_mod2 (5'-AAC RGG ATT AGA TAC CCT GGT AGT CC-3')[5] - Bac908R_mod (5'- CCC GTC AAT TCM TTT GAG TT-3')[6], and (3) Bac908F_mod (5'-AAC TCA AAK GAA TTG ACG GG-3')[6] - Bac1075R (5'-CAC GAG CTG ACG ACA RCC-3')[7]. The thermal cycler protocols were the same as described in the Methods, except that an annealing temperature of 56°C was used for all bacterial assays, and an elongation time of 15s was used for all archaeal assays.

Core descriptions (2012)

Ten Rumohr cores were taken each from M1 and from M5 in March 2012 and described based on sediment color changes and distributions of burrows. All of these cores showed visual signs of an oxidized surface mixed layer (SML), where sediments had a predominantly light brown color. This layer was typically 4±1 cm thick. Below this SML, sediments became predominantly dark grey, presumably due to the accumulation of iron(II) sulfides, while locally still containing light brown streaks, that indicated occasional transport of oxidized surface sediments to deeper layers by macrofauna. This deep mixed layer (DML) extended from approximately 4 to 10 cmbsf at station M1. At M5, the DML was more variable in depth, ranging from ~4-12 cm in the microbiology core, and from ~4 to 7 to ~4 to 20 cmbsf in the other nine cores. All Rumohr cores had worm burrows. These burrows ranged down to 22 cmbsf and 17 cmbsf in the microbiology cores from M1 and M5, respectively. In the other

cores, the burrows ranged down to 15 to 27 cmbsf at M1, and to 14 to 40 cmbsf at M5. Deep-ranging burrows were typically ~1 cm in diameter, ranged in direction from sloping to nearly horizontal, and had rough-textured inner walls with no lining and no signs of oxidation. Macrofauna were observed in two cores: one worm (*Heteromastus filiformis*) was present at 10 cm sediment depth in the core from M1; this core was also used for methane, DIC, and TOC analyses. Furthermore, one small specimen of *Echinocardium cordatum* (diameter ~0.5 cm) was found in surface sediment of the microbiology core from M5. Below the DML, sediments remained dark grey until a second color change, from dark grey back to a lighter brown, occurred further below. The depth of this color transition was highly variable, occurring between 28-48 cmbsf at M1, and between 35-57 cmbsf at M5, and correlates with a strong decrease in microbial sulfate reduction rates between 30 and 50 cmbsf at M1[1, 2], and roughly with the depth of the SMTZ at M5. This deep color transition could be due to decreased sulfate reduction rates and consequentially lower accumulation of iron(II) sulfides in layers below the transition.

The sediment cores from Aarhus Harbor differed markedly from the Aarhus Bay stations in that macrofauna or any signs of macrofaunal activity were absent. Sediments were uniformly black below the top ~1 mm, presumably due to high rates of sulfate reduction and metal sulfide precipitation.

Archaeal and bacterial community compositions at M1

The same DNA extracts from five depths at M1 and the same archaeal and bacteria primer pairs as described above were used for PCR followed by DNA sequencing. Each 50-µL PCR reaction mixture (1st PCR) contained 25 µl 2× KAPA HiFi HotStart ReadyMix (Kapa Biosystems), 0.4 µM of each primer, and 1 µL DNA template. The thermal cycler protocols were (1) enzyme activation and initial denaturation at 95°C for 5 min; (2) 15 cycles of (a)

denaturation at 98°C for 20 sec, (b) primer annealing at 56°C (A1 and A2) and 60°C (A3, B1, B2 and B3) for 30 sec, and (c) elongation at 72°C for 15 sec; (3) final extension at 72°C for 5 min. PCR products were visualized using gel electrophoresis and cleaned using the Agencourt Ampure XP kit (Beckman Coulter, Brea CA). Cleaned PCR products were subjected to a second PCR amplification with barcoded PCR primers (8 bp barcode) in preparation for multiplex amplicon sequencing on an Ion Personal Genome Machine (PGM™) system (Ion Torrent, Life Technologies). PCR protocols with barcode-tagged primers were the same as for the first PCR, except that the 1st PCR product was used as a DNA template, the annealing temperature was 5°C higher, and 27 thermal cycles were used. The barcoded PCR products were visualized using gel electrophoresis and cleaned using the Agencourt Ampure XP kit as for the 1st PCR products. Cleaned PCR products were quantified using the Qubit hs-DS-DNA kit (Invitrogen) on a Qubit® 2.0 Fluorometer (Invitrogen, Life Technologies) reading at 485 nm excitation and 530 nm emission, pooled at equimolar concentrations of each barcoded PCR product, and sequenced using an Ion PGM™ system with one 314 chip and one 316 chip.

Sequence de-multiplexing and bioinformatic processing were performed using Mothur pipelines[8]. Sequences were analyzed individually for each primer pair, since the amplicons covered different regions. Initial quality filtering excluded all sequences that were shorter than 100 bp or contained primer mismatches, homopolymer runs >8 bp, or a mean quality score below 20. Acacia was used to correct amplicon pyrosequencing errors[9]. Uchime was used to detect chimeric sequences[10], which were removed before OTU classification. The high quality sequences were then clustered using an average neighbor algorithm at a 97% sequence similarity cutoff. OTUs with singleton reads were filtered and dropped before further analysis. OTUs were given taxonomic assignments based on full-length sequences and taxonomy references from Silva Release 119

(http://www.mothur.org/wiki/Silva_reference_files). For each primer pair, the libraries generated for each of the three DNA extracts per sediment depth were used to calculate average percentages of each taxonomic group, which were then used in all analyses on archaeal and bacterial community structure.

To compare the community compositions across the different primer pairs and depths analyzed, pairwise sample similarities (Bray Curtis) of archaeal and bacterial sequences were calculated at the species level and visualized using Unweighted Pair Group Method with Arithmetic Mean (UPGMA) trees by software PAST[11].

Supplementary Tables

Supplementary Table 1: Basic information on sampling stations. mbsf, meters below seafloor; SMTZ = sulfate-methane transition zone, defined as the depth interval where the concentration ratio of sulfate to methane ranges from 2:1 to 1:2, and where the highest rates of anaerobic oxidation of methane occur; N/A = not applicable, because this depth was not reached in the cored interval, ND = not determined.

Station	Location	Water Depth (m)	Sampling Time	Core length (mbsf)	Depth interval of SMTZ (mbsf)	Depth of free CH_4 gas (mbsf)
**M21**	56°06.810′N, 10°24.714′E	18.4	Oct. 2009	2.97	N/A	N/A
**M22A**	56°06.745´N, 10°24.880´E	18.8	May 2010	4.82	N/A	N/A
**M24**	56°06.713′N, 10°24.981′E	18.8	Oct. 2009	5.80	3.36-4.31	N/A
**M26**	56°06.693′N, 10°25.029′E	19.1	Oct. 2009	5.54	3.04-3.78	7.04
**M27A**	56°06.679´N, 10°25.083´E	19.1	May 2010	7.12	2.62-2.90	4.70
**M29A**	56°06.645´N, 10°25.175´E	19.3	May 2010	7.20	2.20-2.75	4.45
**M1**	56°09.167′N, 10°19.333′E	15.0	Oct. 2009	10.85	1.40-1.60	4.00
			May 2010	9.20	1.94-2.03	ND
			March 2012	0.62	N/A	N/A
**M5**	56°06.333′N, 10°27.800′E	27.0	May 2010	6.70	0.47-0.58	1.00
			March 2012	0.81	0.48-0.56	N/A
**AHB**	56°08.324′N, 10°12.877′E	~2.0	July 2012	0.15	N/A	N/A

Supplementary Table 2: Summary of average bacterial and archaeal 16S gene abundances and BARs across 8 stations and 4 biogeochemical zones. Archaeal and bacterial 16S gene copy numbers are shown in units of 10^8 copies cm^{-3} of wet sediment.

**Zones**		**Bioturbation zone**			**Sulfate zone**			**SMTZ**			**Methane zone**		
**Stations**		**mean±std.**	**range**	***n***	**mean±std.**	**range**	***n***	**mean±std.**	**range**	***n***	**mean±std.**	**range**	***n***
**M21**	**Archaea**	1.44±0.89	0.21-2.30	*3*	1.82±1.67	0.23-4.57	*4*	0.27	0.06	*2*	/	/	*0*
	**Bacteria**	19.68±4.73	13.10-24.01		3.23±2.95	0.10-7.73		0.09	0.00		/	/	
	**BAR**	28.33±24.13	10.42-62.43		1.48±0.70	0.43-2.37		0.33	0.06		/	/	
**M22A**	**Archaea**	1.30±0.72	0.58-2.02	*2*	2.12±1.75	0.25-4.95	*5*	0.73	0.73	*1*	/	/	*0*
	**Bacteria**	18.59±1.43	17.16-20.02		4.32±3.54	0.38-9.86		0.61	0.61		/	/	
	**BAR**	21.52±13.01	8.52-34.53		1.97±0.33	1.35-2.24		0.84	0.84		/	/	
**M24**	**Archaea**	2.30±1.34	0.91-4.10	*3*	1.80±1.30	0.32-3.40	*4*	0.13±0.03	0.10-0.16	*3*	0.16±0.02	0.14-0.18	*2*
	**Bacteria**	30.87±5.78	24.70-38.61		4.07±3.30	0.58-7.55		0.15±0.02	0.12-0.17		0.11±0.00	0.11-0.12	
	**BAR**	21.30±15.40	6.02-42.37		2.00±0.54	1.30-2.78		1.16±0.09	1.05-1.25		0.71±0.12	0.59-0.84	
**M26**	**Archaea**	0.78±0.21	0.55-1.05	*3*	1.77±1.46	0.40-4.12	*4*	0.63±0.23	0.34-0.91	*3*	0.36±0.10	0.28-0.50	*3*
	**Bacteria**	35.79±11.70	25.35-52.13		3.47±2.78	0.62-8.05		0.49±0.16	0.28-0.66		0.26±0.08	0.19-0.37	
	**BAR**	46.17±8.14	34.95-53.99		2.23±1.17	1.20-4.20		0.78±0.04	0.73-0.80		0.71±0.06	0.62-0.78	
**M27A**	**Archaea**	2.09±0.96	1.14-3.05	*2*	2.51±1.93	0.13-4.30	*5*	0.41±0.22	0.22-0.72	*3*	0.19±0.04	0.14-0.23	*4*
	**Bacteria**	26.44±4.81	21.63-31.25		5.52±4.71	0.38-13.00		0.57±0.12	0.45-0.73		0.18±0.10	0.07-0.32	
	**BAR**	17.30±10.21	7.09-27.50		2.53±0.88	1.66-4.00		1.63±0.53	1.02-2.32		0.86±0.36	0.50-1.43	
**M29A**	**Archaea**	3.04±2.09	0.95-5.13	*2*	3.29±2.66	0.10-6.81	*5*	0.59±0.33	0.27-1.05	*3*	0.48±0.24	0.24-0.87	*4*
	**Bacteria**	36.80±7.59	29.21-44.39		6.40±4.91	0.29-12.31		0.77±0.06	0.71-0.86		0.52±0.17	0.25-0.70	
	**BAR**	26.24±20.55	5.70-46.79		2.35±0.52	1.81-2.98		1.72±0.80	0.82-2.76		1.22±0.49	0.71-2.02	
**M1(2009)**	**Archaea**	0.89±0.13	0.77-1.02	*2*	2.89±0.63	2.04-3.75	*4*	3.11±1.76	1.18-5.43	*3*	1.40±0.21	1.23-1.76	*4*
	**Bacteria**	43.84±6.85	36.99-50.70		5.11±2.36	5.41-34.43		3.01±1.85	1.39-5.59		1.06±0.27	0.80-1.46	
	**BAR**	51.17±14.98	36.19-66.15		1.78±0.69	0.94-2.53		0.99±0.18	0.75-1.18		0.78±0.27	0.46-1.19	

Zones / Stations		Bioturbation zone			Sulfate zone			SMTZ			Methane zone		
		**mean±std.**	**range**	***n***	**mean±std.**	**range**	***n***	**mean±std.**	**range**	***n***	**mean±std.**	**range**	***n***
**M5(2010)**	**Archaea**	/	/	*0*	1.25±1.13	0.37-2.84	*3*	1.46±0.12	1.37-1.64	*3*	0.83±0.51	0.24-1.48	*3*
	**Bacteria**	/	/		3.81±3.71	1.15-9.05		6.01±2.71	3.42-9.75		2.56±1.79	0.42-4.80	
	**BAR**	/	/		2.85±2.49	2.22-3.19		4.23±5.30	2.09-7.06		2.74±0.71	1.74-3.26	
**M1(2012)**	**Archaea**	2.72±0.58	1.98-3.64	*7*	3.05±1.12	1.41-5.52	*12*	/	/	*0*	/	/	*0*
	**Bacteria**	25.22±17.55	6.25-56.81		5.97±2.01	4.01-11.60		/	/		/	/	
	**BAR**	8.90±6.18	2.65-21.92		2.08±0.56	1.33-2.95		/	/		/	/	
**M5(2012)**	**Archaea**	2.57±0.77	1.32-3.52	*7*	3.63±1.07	1.95-5.30	*9*	3.41±1.83	1.65-5.93	*3*	3.55±1.22	2.27-5.58	*5*
	**Bacteria**	18.05±12.00	7.80-44.97		8.00±1.92	4.73-10.44		6.34±3.25	3.08-10.77		4.94±1.35	3.67-6.98	
	**BAR**	7.81±5.31	2.71-16.39		2.27±0.32	1.76-2.78		1.88±0.06	1.82-1.95		1.43±0.14	1.25-1.63	
**Overall**	**Archaea**	2.10±1.18	0.21-5.13	*31*	2.65±1.68	0.10-6.81	*55*	1.27±1.51	0.10-5.93	*24*	1.20±1.37	0.14-5.58	*25*
	**Bacteria**	26.24±13.80	6.25-56.81		5.54±3.50	0.10-13.00		2.20±2.94	0.08-10.77		1.61±2.01	0.07-6.98	
	**BAR**	20.54±18.97	2.65-66.15		2.15±0.70	0.43-4.20		1.61±1.36	0.28-7.06		1.21±0.72	0.46-3.26	

Supplementary Table 3: List of the dominant sediment macrofauna and their size, burial depth and feeding mode in central Aarhus Bay.

Type	Species	Adult size (cm)	Burial depth	Feeding mode and depth	References
Bivalves	*Abra alba*	2.5	0.5-2.5 cm*	Mainly surface deposit feeder, but also suspension feeder	[12, 13]
	Mysella bidendata	0.3	Surface, burrows of other species, crevices	Surface deposit and suspension feeder; mainly deposit feeder in Aarhus Bay	[14-17]
	Corbula gibba	1.5	1-2 cm	Surface suspension feeder	[18, 19]
	Macoma calcarea	3.5-5	0-10 cm	Surface deposit feeder, suspension feeder	[20]
Polychaetes	*Terebellides stroemi*	7.5	Tube extends to several cm below sediment surface	Sedentary surface deposit feeder that builds a tube, preference for small, low-gravity particles	[21]
	Nephtys ciliate, Nephtys sp.	10-30	Commonly to 5-10 cm, but also deeper	Predator, scavenger, deposit feeder; builds temporary burrows; biodiffusor	[22-24]
	Heteromastus filiformis	Length: 10, width: 0.1	0-30 cm	Subsurface deposit feeder with upward conveyor belt transport, builds burrows	[13, 14, 21]
	Pectinaria sp.	4.5	Tubes extend to 5 cm depth	(Mainly subsurface) deposit feeder with upward conveyor belt transport, builds tube	[14, 25]
Echinoderms	*Ophiura albida*	Disc: 1.5, arms: 6	Stays at surface	Surface predator, scavenger, deposit feeder	[15]
	Echinocardium cordatum	5	0-15 cm	Surface deposit feeder, surface to subsurface burrower	[26]

*inferred from close relative *Abra nitida*

Supplementary Figures

Supplementary Figure 1: Map of Aarhus Bay, Denmark, with locations of sampling stations (modified from reference #27). The gray shades indicate the shallowest depth at which free gas bubbles appear.

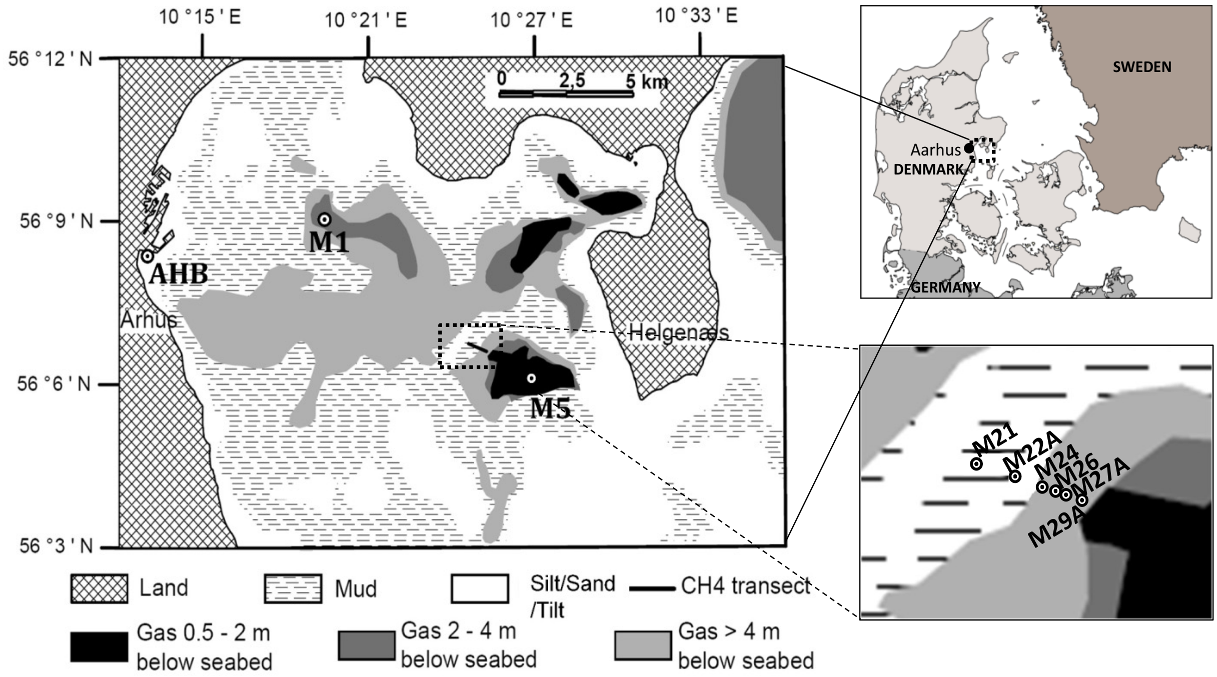

Supplementary Figure 2: Depth distributions of archaeal and bacterial 16S gene abundances across the CH_4 transect in Aarhus Bay (M21-M29A), and at Stations M1 and M5. The lower figures show data from the upper figures at high resolution for the top 0.5 m. Grey shaded areas mark the depth interval of the SMTZ (also see Supplementary Table 1). Sediments above belong to the sulfate zone (SZ), sediments below belong to the methane zone (MZ; also see labeling in figure for M24). The gray circle at M21 indicates samples from an organic-poor glacial sand layer at the bottom of M21. The SMTZ and MZ were not reached by coring at M21 and M22A.

Supplementary Figure 2

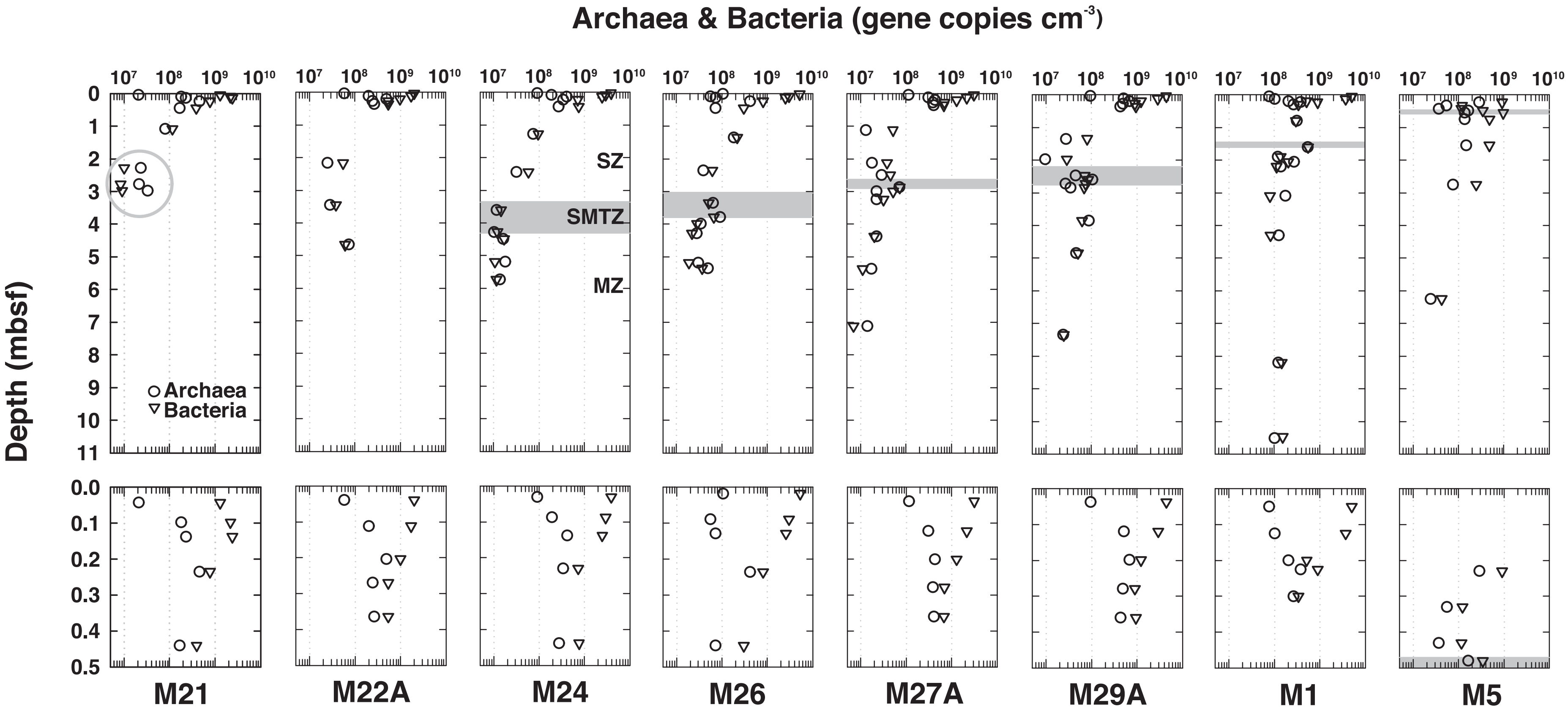

Supplementary Figure 3: Fine-scale geochemical profiles at Stations M1 (top) and M5 (bottom). (**a, b**) methane concentrations; (**c, d**) total organic carbon (TOC) in weight % (wt%); (**e, f**) porosity; (**g, h**) density. The horizontal grey dashed line spanning all panels marks the depth to which significant sediment reworking was detected (0.14 mbsf). The grey zone at M5 marks the SMTZ. All data were obtained from Rumohr cores collected in March 2012.

Supplementary Figure 3

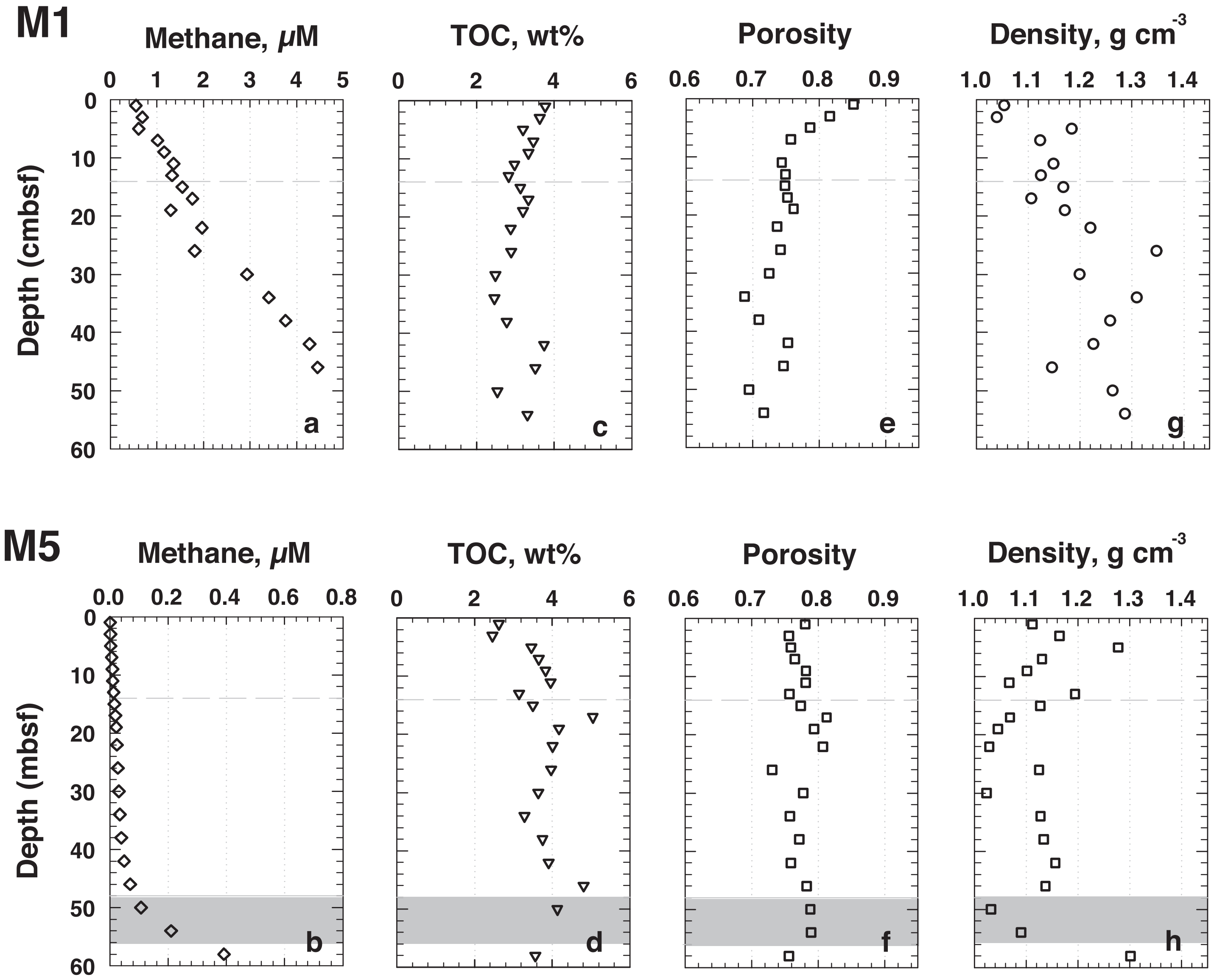

Supplementary Figure 4: (a) Archaeal and bacterial 16S gene abundances, and (b) BARs in surface sediment at the bioturbation-free control site in Aarhus Harbor (AHB) compared to stations in Aarhus Bay.

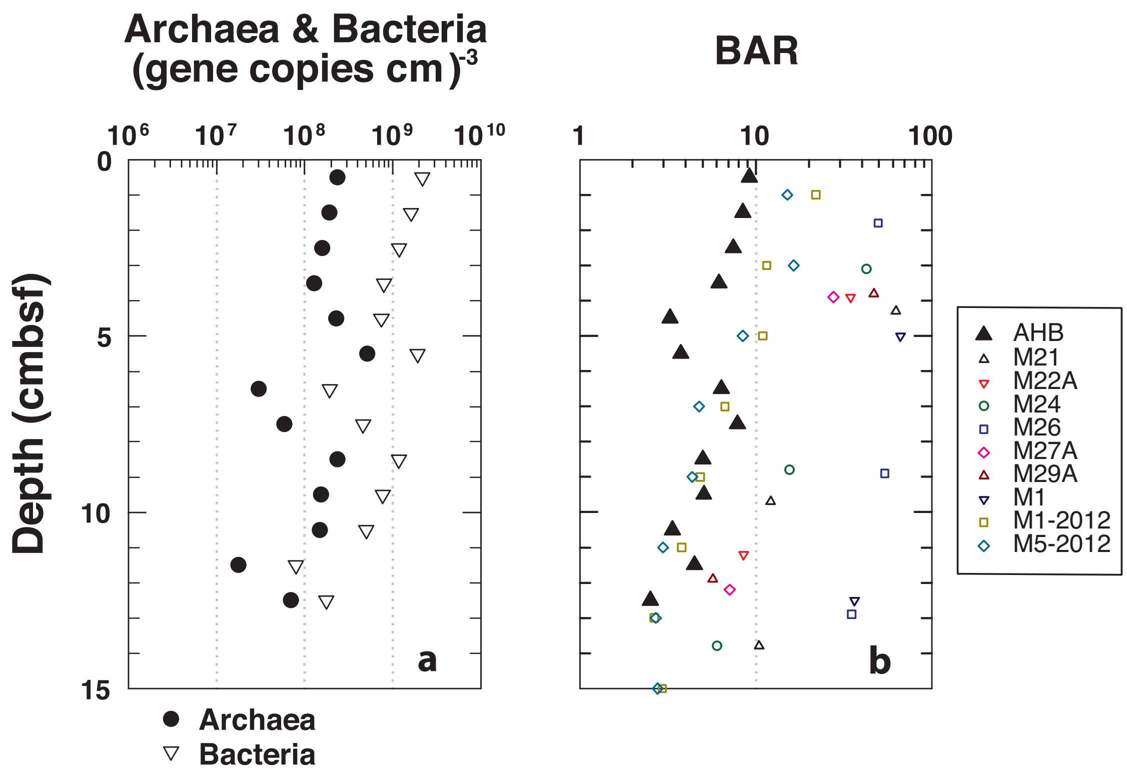

Supplementary Figure 5: Scatter plots illustrating the relationships between TOC, TN, C:N, δ^{13}C-TOC, and δ^{15}N-TN and BAR, archaeal 16S gene abundances, and bacterial 16S gene abundances. (**a**) Samples spanning the bioturbation zone; (**b**) samples spanning sediments underlying the bioturbation zone; (**c**) samples spanning sediments underlying the bioturbation zone excluding all terrestrial samples. R^2-values are for the trendline shown in each plot. Statistical significance of the correlations was determined using a Spearman's Rank Correlation test (ns = not significant; asterisks indicate statistical significance with the number of asterisks indicating the level of significance: * for $p<0.05$, ** for $p<0.01$, and *** for $p<0.001$).

Supplementary Fig 5a

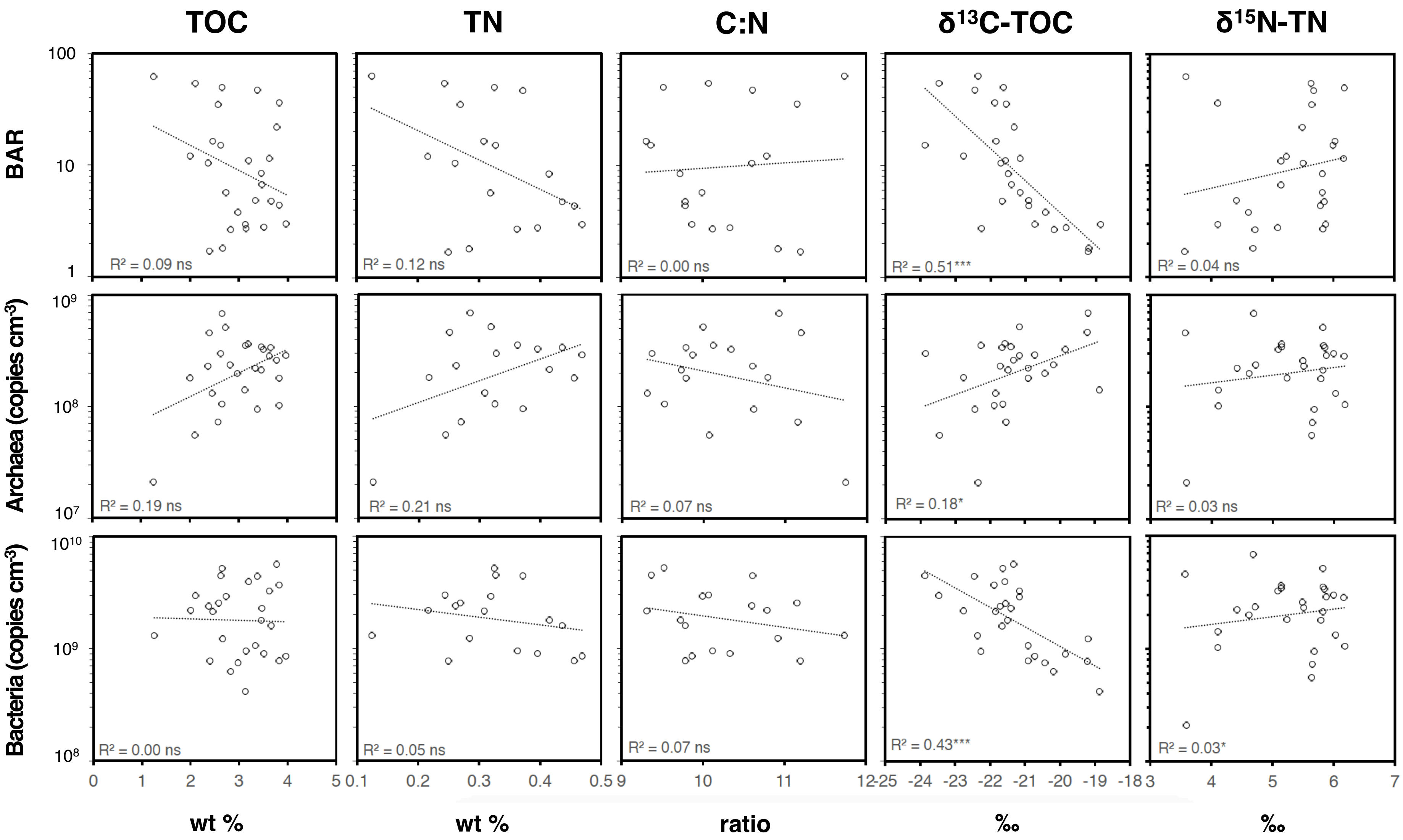

Supplementary Fig. 5b

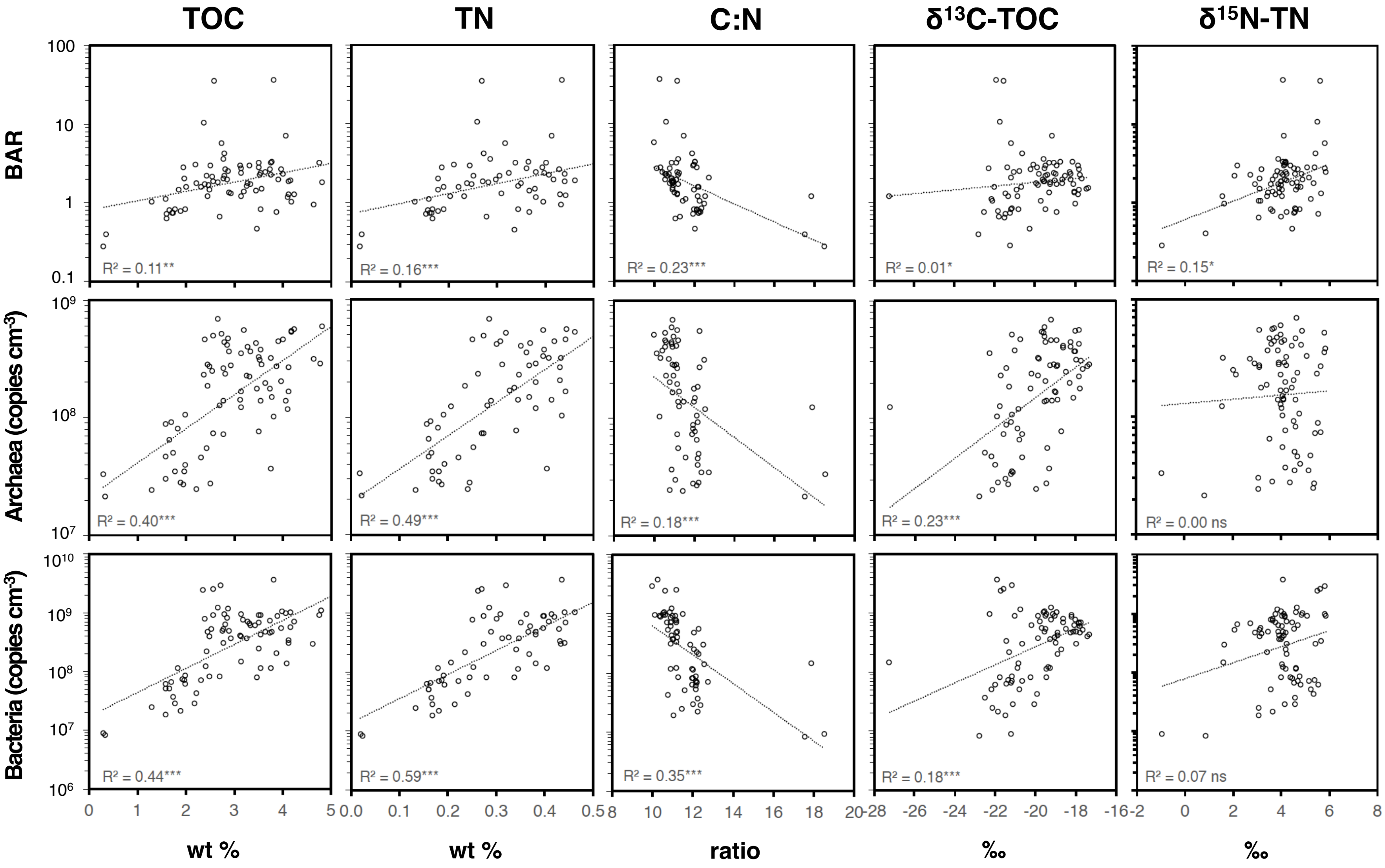

Supplementary Fig. 5c

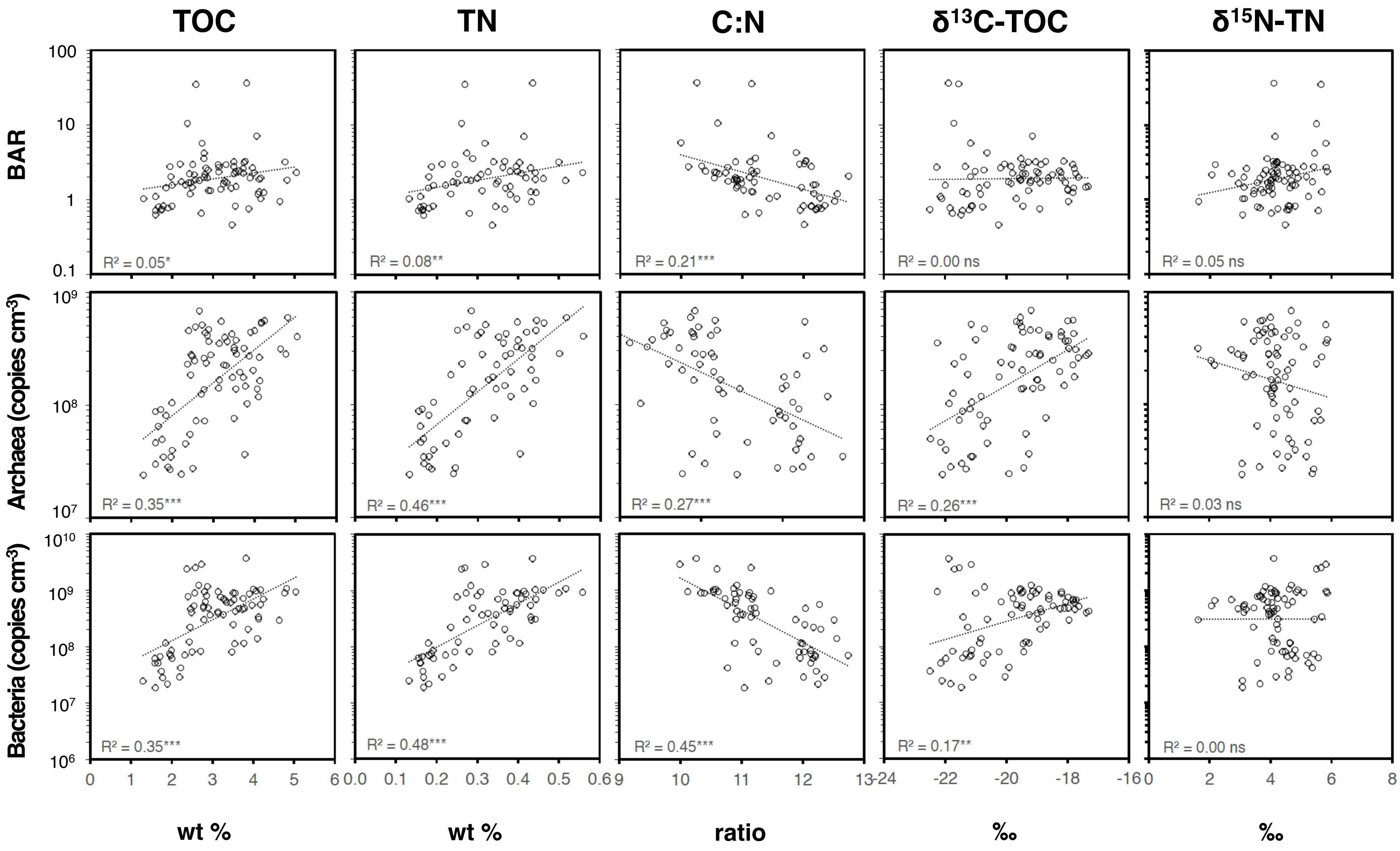

Supplementary Figure 6: (a) Archaeal and (b) bacterial 16S gene abundances yielded by different primer pairs on three extraction replicates per depth at M1; (c) box plots of BARs. (a, b) Three qPCR replicates were applied on each of the three extraction replicates and primer combinations. The average values for each extraction replicate and primer combination are plotted. (c) Box plots were based on BARs calculated from each of the bacterial and archaeal primer pair combinations (3 archaeal × 3 bacterial primer pairs, thus n=9). Blue lines inside boxes indicate average values, black lines indicate median values. Boxes indicate 25% and 75% confidence intervals. Error bars indicate 10 and 90% confidence intervals. The primer combinations Arc 806F-Arc 958R and Bac 8Fmod-Bac 338Rabc were used throughout this study.

Supplementary Figure 6

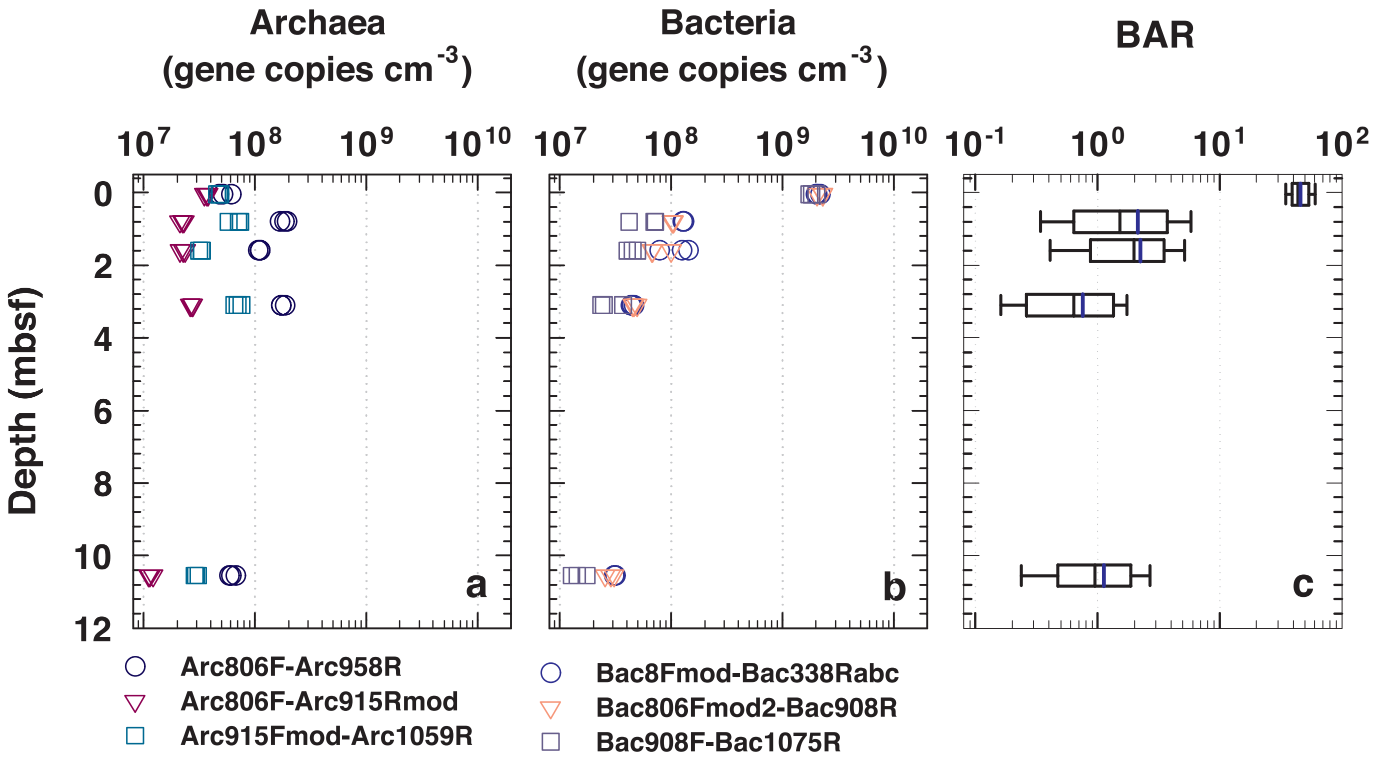

Supplementary Figure 7: Cell abundance profiles at M22A, M27A, M29A, M1, and M5 determined by fluorescence microscopy. The grey dashed line spanning across the lower panels indicates the minimum depth of sediment reworking. The grey shaded areas mark the SMTZ. Data from M1 and M5 were previously published[28]. Note: the SMTZ at M1 was ~0.5m deeper in the M1 core from May 2010 (see below) than in the M1 core from October 2009, which was used for DNA extractions (Supplementary Table 1 and Supplementary Figure 2).

Supplementary Figure 7

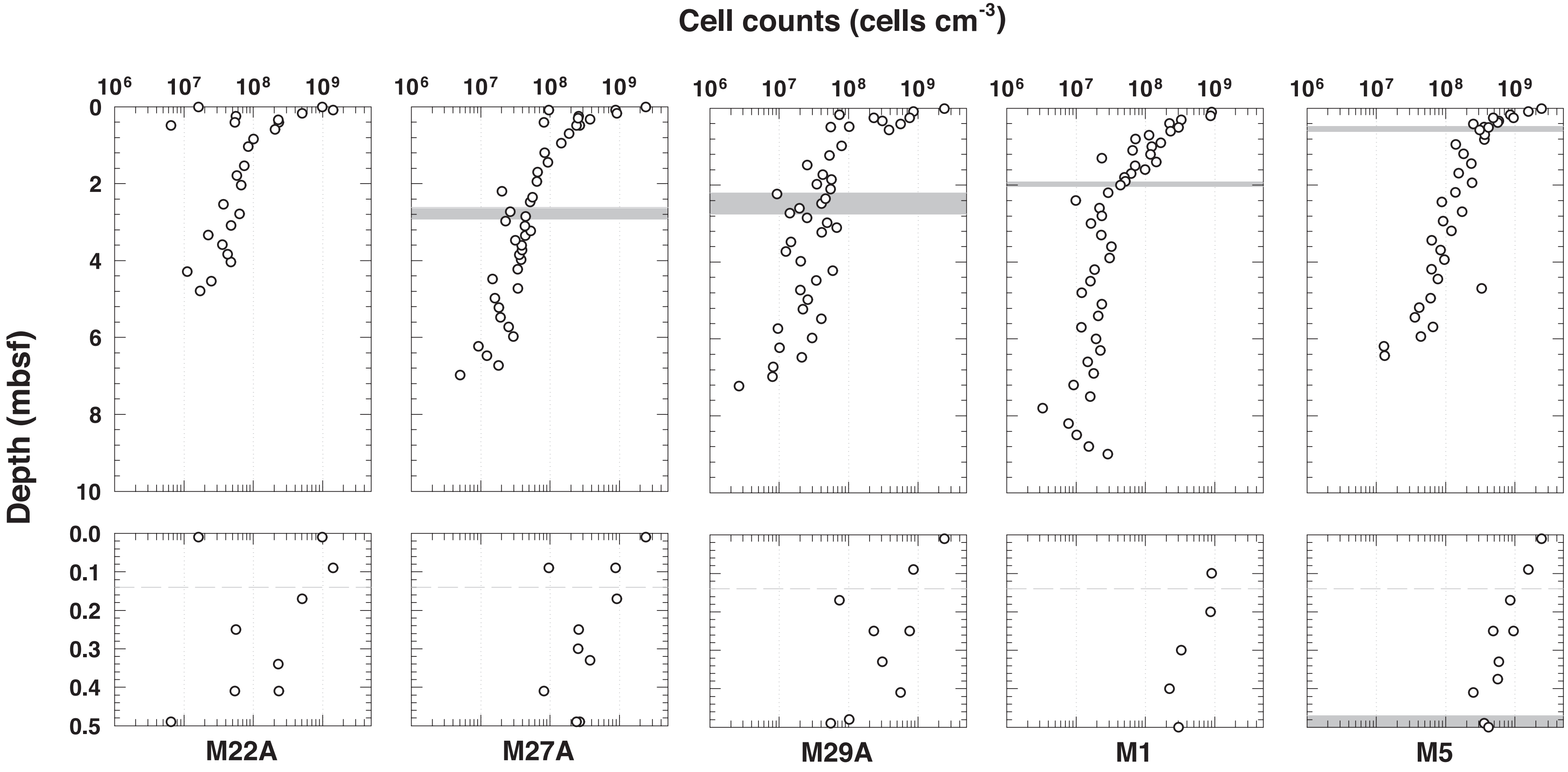

Supplementary Figure 8: Cell abundances and 16S gene copy numbers per cell in Aarhus Bay. (a) Depth distribution of cell abundances at M22A, M27A, M29A, M1, and M5 compiled from Supplementary Fig. 7. Cell abundances from M1 and M5 were previously published[28]. The grey solid line is the regression line (Log cell abundance = 8.05 − 0.68 Log depth) and the grey dashed lines indicate the 95% lower and upper prediction limits based on prokaryotic cell abundances in subsurface sediments at 106 locations[29]. (**b**) Depth distributions of 16S gene copy numbers per cell at M22A, M27A, M29A, and M5. The mean copy numbers per cell (averages±standard) at individual stations are: M22A, 4.3±4.2; M27, 2.2±1.4; M29A, 4.2±2.5; M5, 2.0±1.4 copies cell^{-1}. Cell counts and 16S rRNA gene copy numbers were obtained from the same cores (M22A, M27A, M29A), or from parallel cores taken on the same sampling date (M5). We omit data from M1, where cell counts and gene copy numbers were obtained from cores collected on different dates (cell counts: May 2010; DNA: October 2009) and differed clearly in sulfate concentration profiles and the depth of the SMTZ (Supplementary Table 1).

Supplementary Figure 8

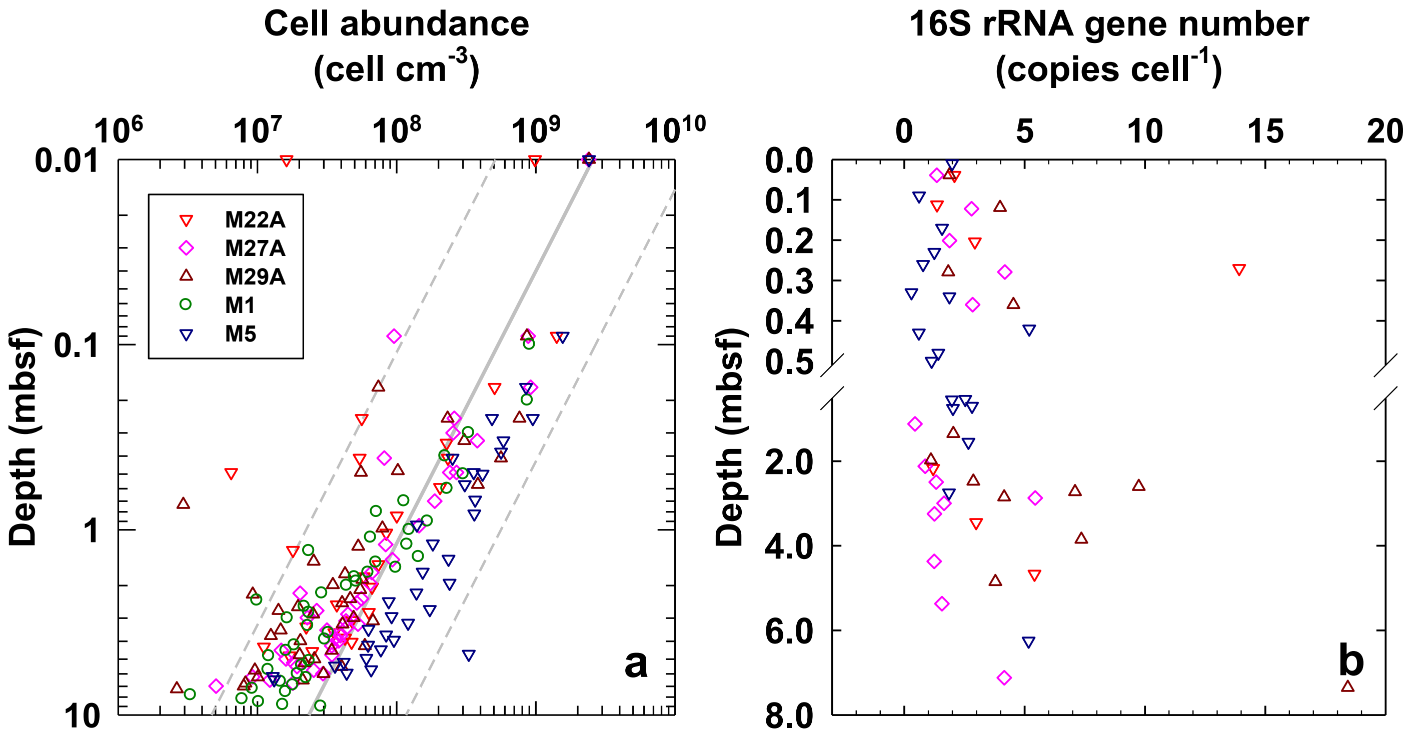

Supplementary Figure 9: UPGMA trees (OTU level) for archaeal and bacterial communities among five depths at M1. The UPGMA tree for Archaea was calculated from the archaeal community compositions generated by archaeal primer pairs (A1: Arc806F - Arc958R, A2: Arc806F - Arc915Rmod; A3: Arc915Fmod - Arc1059R). The UPGMA tree for Bacteria was calculated from the bacterial community compositions generated by bacterial primer pairs (B1: Bac8F - Bac338Rabc, B2: Bac806F_mod2 - Bac908R; B3: Bac908F - Bac1075R). Algorithm: Paired group; similarity measurement: Bray-Curtis; number of bootstraps: 1,000. The depths represent the following zones: 5 cmbsf: bioturbation zone, 80 cmbsf: sulfate zone, 160 cmbsf: SMTZ, 310 cmbsf: shallow methane zone, 1055 cmbsf: freshwater peat layer in deep methane zone.

Supplementary Figure 9

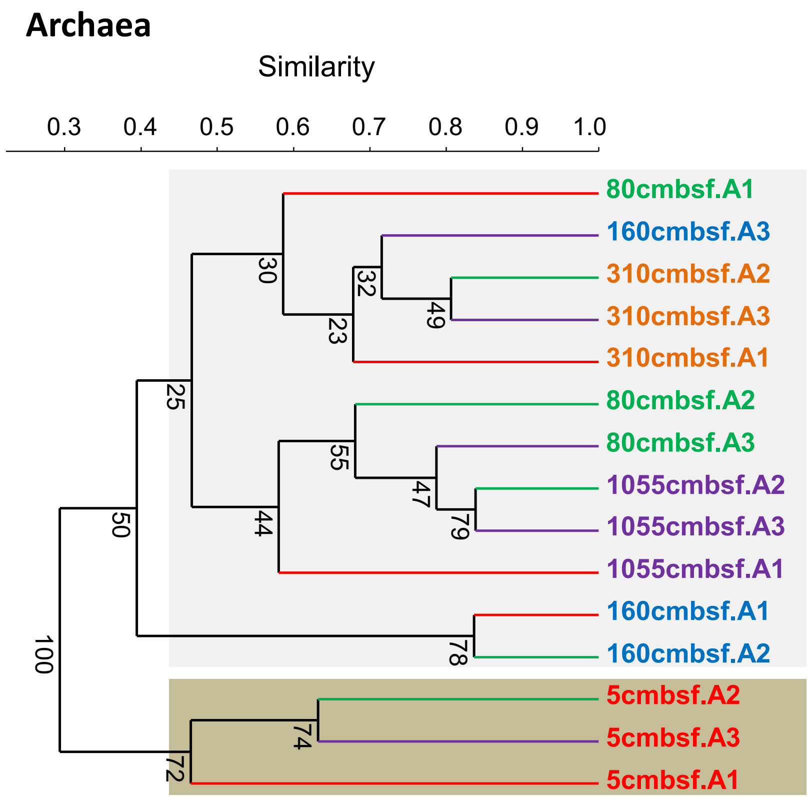

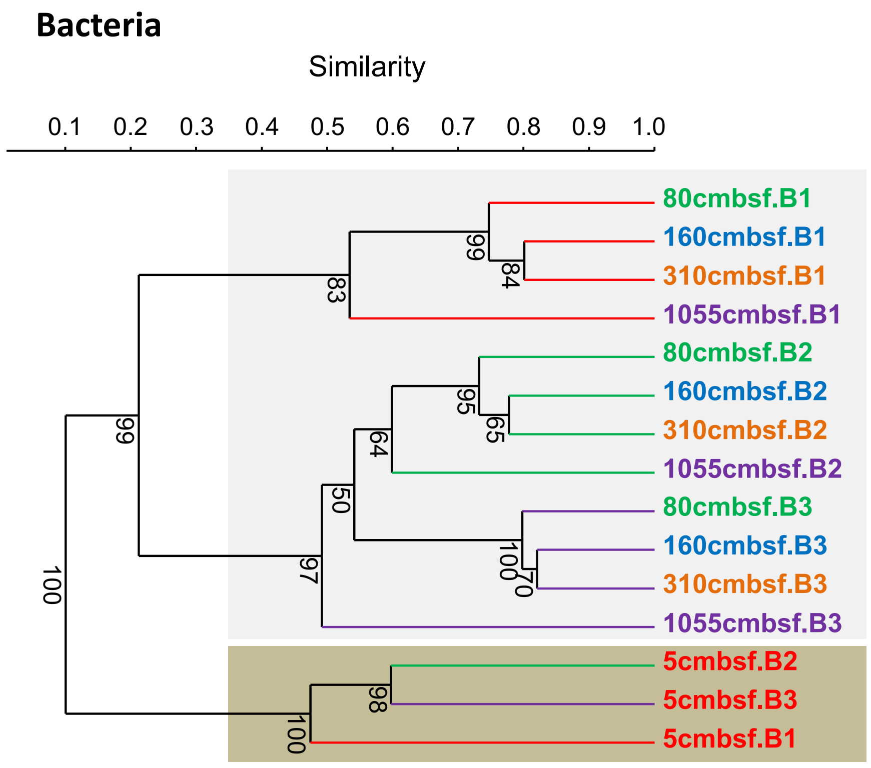

Supplementary References

1. Holmkvist, L., Ferdelman, T. G. & Jørgensen, B. B. A cryptic sulfur cycle driven by iron in the methane zone of marine sediment (Aarhus Bay, Denmark). *Geochim. Cosmochim. Acta* **75**, 3581-3599 (2011).

2. Leloup, J.*, et al.* Sulfate-reducing bacteria in marine sediment (Aarhus Bay, Denmark): Abundance and diversity related to geochemical zonation. *Environ. Microbiol.* **11**, 1278-1291 (2009).

3. Cadillo-Quiroz, H.*, et al.* Vertical profiles of methanogenesis and methanogens in two contrasting acidic peatlands in central New York State, USA. *Environ. Microbiol.* **8**, 1428-1440 (2006).

4. Yu, Y., Lee, C., Kim, J. & Hwang, S. Group-specific primer and probe sets to detect methanogenic communities using quantitative real-time polymerase chain reaction. *Biotechnol. Bioeng.* **89**, 670-679 (2005).

5. Nadkarni, M., Martin, F. E., Jacques, N. & Hunter, N. Determination of bacterial load by real-time PCR using a broad-range (universal) probe and primers set. *Microbiology* **148**, 257-266 (2002).

6. Lever, M. A. , Torti, A., Eickenbusch, P., Michaud, A. B., Šantl-Temkiv, T. & Jørgensen, B. B.A modular method for the extraction of DNA and RNA, and the separation of DNA pools from diverse environmental sample types. *Front. Microbiol.* **6**, (2015).

7. Ohkuma, M. & Kudo, T. Phylogenetic analysis of the symbiotic intestinal microflora of the termite *Cryptotermes domesticus*. *FEMS Microbiol. Lett.* **164**, 389-395 (1998).

8. Schloss, P. D.*, et al.* Introducing mothur: open-source, platform-independent, community-supported software for describing and comparing microbial communities. *Appl. Environ. Microbiol.* **75**, 7537-7541 (2009).

9. Bragg, L., Stone, G., Imelfort, M., Hugenholtz, P. & Tyson, G. W. Fast, accurate error-correction of amplicon pyrosequences using Acacia. *Nature Meth.* **9**, 425-426 (2012).

10. Edgar, R. C., Haas, B. J., Clemente, J. C., Quince, C. & Knight, R. UCHIME improves sensitivity and speed of chimera detection. *Bioinformatics* **27**, 2194-2200 (2011).

11. Hammer, Ø., Harper, D. A. T. & Ryan, P. D. Paleontological statistics software package for education and data analysis. *Palaeontol. Electron.* **4**, 9-18 (2001).

12. Maire, O., Duchene, J. C., Rosenberg, R., de Mendonca Jr., J.B. & Gremare, A. Effects of food availability on sediment reworking in *Abra ovata* and *A. nitida*. *Mar. Ecol. Prog. Ser.* **319**, 135-153 (2006).

13. Thayer, C. W. Sediment-mediated biological disturbance and the evolution of marine benthos. In: *Biotic Interactions in Recent and Fossil Benthic Communities* (eds Tevesz MJS). Plenum Press (1983).

14. Dauwe, B., Herman, P. M. J. & Heip, C. H. R. Community structure and bioturbation potential of macrofauna at four North Sea stations with contrasting food supply. *Mar. Ecol. Prog. Ser.* **173**, 67-83 (1998).

15. Fallesen, G. *The ecology of macrozoobenthos in Aarhus Bay, Denmark* PhD thesis, University of Stirling, (1994).

16. Josefson, A. B. & Rosenberg, R. Long-term soft-bottom faunal changes in three shallow fjords, West Sweden. *Neth. J. Sea. Res.* **22**, 149-159 (1988).

17. O'Foighil, D., McGrath, D., Conneely, M. E., Keegan, B. F. & Costelloe, M. Population dynamics and reproduction of *Mysella bidentata* (Bivalvia: Galeommatacea) in Galway Bay, Irish west coast. *Mar. Biol.* **81**, 283-291 (1984).

18. Kiørboe, T. & Møhlenberg, F. Particle selection in suspension-seeding bivalves. *Mar. Ecol. Prog. Ser.* **5**, 291-296 (1981).

19. Yonge, C. M. On the habits and adaptations of *Aloidis* (*Corbula*) *gibba*. *J. Mar. Biol. Assoc. U.K.* **26**, 358-376 (1946).

20. Richman, S. E. & Lovvorn, J. R. Effects of clam species dominance on nutrient and energy acquisition by spectacled eiders in the Bering Sea. *Mar. Ecol. Prog. Ser.* **261**, 283-297 (2003).

21. Gogina, M. & Zettler, M. L. Diversity and distribution of benthic macrofauna in the Baltic Sea. *J. Sea Res.* **64**, 313-321 (2010).

22. Clark, R. B. Observations on the food of *Nephtys*. *Limnol. Oceanogr.* **7**, 380-385 (1962).

23. Michaud, E., Aller, R. C. & Stora, G. Sedimentary organic matter distributions, burrowing activity, and biogeochemical cycling: Natural patterns and experimental artifacts. *Estuar. Coast. Shelf Sci.* **90**, 21-34 (2010).

24. Pedersen, T. F. & Munkegade, N. Metabolic adaptations to hypoxia of two species of Polychaeta, *Nephtys ciliata* and *Nephtys hombergii*. *Mar. Biol.* **161**, 213-215 (1991).

25. Dobbs, F. & Scholly, T. Sediment processing and selective feeding by *Pectinaria koreni* (Polychaeta: Pectinariidae) *Mar. Ecol. Prog. Ser.* **29**, 165-176 (1986).

26. Buchanan, J. B. The biology of *Echinocardium cordatum* [Echinodermata: Spatangoidea] from different habitats. *J. Mar. Biol. Assoc. U.K.* **46**, 97-114 (1966).

27. Jensen, J. B. & Bennike, O. Geological setting as background for methane distribution in Holocene mud deposits, Aarhus Bay, Denmark. *Cont. Shelf Res.* **29**, 775-784 (2009).

28. Langerhuus, A. T., *et al.* Endospore abundance and d:l-amino acid modeling of bacterial turnover in holocene marine sediment (Aarhus Bay). *Geochim. Cosmochim. Acta* **99**, 87-99 (2012).

29. Parkes, R. J., *et al.* A review of prokaryotic populations and processes in sub-seafloor sediments, including biosphere: Geosphere interactions. *Mar. Geol.* **352**, 409-425 (2014).
